# Regulatory T cells in cancer and inflammation

**DOI:** 10.1038/s41392-026-02584-w

**Published:** 2026-06-01

**Authors:** Haoyi Yang, Huafeng Zhang, Ni Xia, Xiang Cheng

**Affiliations:** 1 Department of Cardiology, Union Hospital, Tongji Medical College, Huazhong University of Science and Technology, Wuhan, Hubei China; 2 Hubei Key Laboratory of Biological Targeted Therapy, Union Hospital, Tongji Medical College, Huazhong University of Science and Technology, Wuhan, Hubei China; 3 Hubei Provincial Engineering Research Center of Immunological Diagnosis and Therapy for Cardiovascular Diseases, Union Hospital, Tongji Medical College, Huazhong University of Science and Technology, Wuhan, China; 4 Key Laboratory of Biological Targeted Therapy (Huazhong University of Science and Technology), Ministry of Education, Wuhan, Hubei China; 5 Department of Pathology, School of Basic Medicine, Tongji Medical College, Huazhong University of Science and Technology, Wuhan, Hubei China

**Keywords:** Tumour immunology, Inflammation, Lymphocytes, Immunotherapy, Immunological disorders

## Abstract

As immunoregulatory cells, regulatory T cells (Tregs) play pivotal roles in maintaining immune tolerance and preventing autoimmunity. However, Tregs exhibit distinct functions across different diseases. In cancer, Tregs are most likely to suppress antitumor immune responses and promote tumor immune evasion, whereas in inflammatory diseases, functionally competent Tregs mitigate excessive immune activation and facilitate tissue repair. Notably, dysfunctional Tregs lead to persistent inflammation and progression to chronic disease. Therefore, targeting Tregs has emerged as an attractive immunotherapeutic strategy for both cancer and inflammatory disorders. Recent studies have shown that Tregs exhibit instability and plasticity under specific conditions, allowing them to shift between functional and dysfunctional states. A comprehensive understanding of the dynamic changes in Tregs and their regulatory mechanisms in diverse pathological contexts is highly important. In this review, we summarize the dual roles of Tregs in cancer and various inflammatory diseases. We explore the signaling pathways and molecular mechanisms underlying their biological characteristics, with a particular focus on how microenvironmental cues shape Treg behavior. Additionally, we discuss recent advances in Treg-targeted therapies in these disease contexts. Overall, this review greatly advances our understanding of the roles of Tregs in cancer and inflammation and helps inform the development of more precise and effective therapeutic strategies.

## Introduction

Tregs constitute a subset of CD4^+^ T lymphocytes characterized by high expression of interleukin-2 receptor alpha (IL2RA, CD25) and the lineage-defining transcription factor forkhead box protein P3 (Foxp3).^1,2^ Tregs are conventionally identified as a group of T cells that perform immunosuppressive functions. Under physiological conditions, they are crucial for maintaining immune tolerance to self-antigens and controlling excessive immune responses that might be harmful to the host.^3,4^ In addition to their conventional immune regulatory functions, Tregs also perform tissue-specific functions, such as facilitating tissue repair following injury and regulating metabolism.^5,6^

However, under certain pathological conditions, Tregs display marked heterogeneity and may exert detrimental effects. For example, in tumors, Tregs frequently accumulate within the tumor microenvironment (TME), where they suppress antitumor immune responses, and may correlate with poor prognosis and reduce the efficacy of various anticancer therapies.^7^ In contrast, in many inflammatory diseases, Tregs play crucial roles in controlling aberrant immune activation and promoting tissue repair, but their dysfunction or instability may lead to uncontrolled inflammation and disease progression.^8–10^ These contrasting roles underscore the need to understand Tregs in a disease-specific manner. Rather than being a functionally stable population, Tregs exhibit significant plasticity and instability modulated by intrinsic and extrinsic factors. This is characterized by either downregulated or unchanged Foxp3 expression, accompanied by the expression of transcription factors and effector molecules associated with other T-cell subsets.^11^ This dynamic behavior presents both challenges and opportunities for Treg-related research. On the one hand, understanding the roles of Tregs across various diseases is complex; on the other hand, this finding offers potential targets for therapeutic intervention by modulating Treg stability: destabilizing Tregs in tumors may enhance antitumor immunity, while stabilizing Tregs in inflamed tissues may restore immune tolerance and resolve pathology.^12,13^

Over the past decade, the rapid advancement of emerging technologies—particularly single-cell sequencing and multiomics approaches—has provided significant insights into the fine details of Treg phenotypes, functions, and their dynamic changes, particularly regarding Treg heterogeneity in different disease microenvironments.^14,15^ This review begins with a brief introduction of Treg classification, differentiation, and the molecular biology underlying their functions. We then focus on recent discoveries concerning Tregs in cancer and inflammatory diseases, along with the signaling pathways and regulatory mechanisms involved. These insights are instrumental in informing and refining future Treg-targeted immunotherapeutic strategies.

## Basic concepts of Tregs

### Classification and differentiation of Tregs

Tregs are classified into two main types according to their origin: thymus-derived Tregs (tTregs) and peripheral-derived Tregs (pTregs), both of which typically express Foxp3.^16^ However, distinguishing tTregs from pTregs remains challenging and controversial.^17^ Some studies suggest that the expression of neuropilin-1 (Nrp1) and Helios may help distinguish tTregs from pTregs in mice, with tTregs tending to express higher levels of both markers.^18,19^ Furthermore, no definitive markers have been identified to differentiate these subsets in humans.^9^ Moreover, induced Tregs (iTregs) can be generated in vitro by stimulating naïve CD4⁺ T cells via the T-cell receptor (TCR) in the presence of transforming growth factor-β (TGF-β) and interleukin-2 (IL-2) to induce Foxp3 expression.^17,20^ Although iTregs have potential for use in adoptive cell therapy (ACT), this review focuses on natural Tregs (tTregs/pTregs) and does not extensively discuss iTregs.

**Fig. 1:**
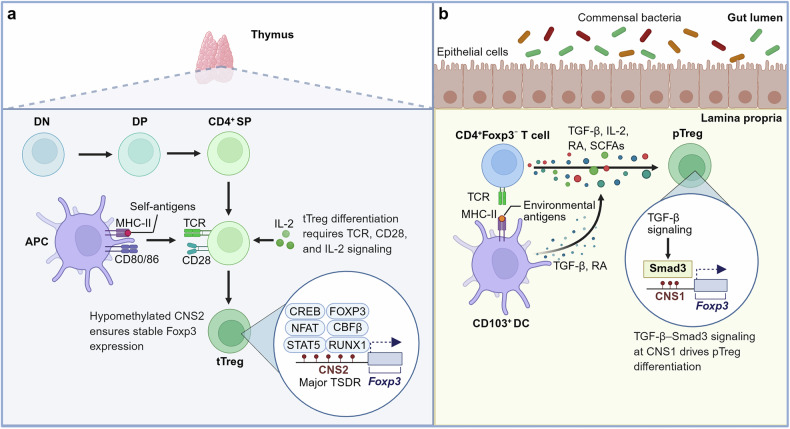
Differentiation of tTregs and pTregs. The peripheral Treg pool comprises tTregs and pTregs, which undergo different differentiation processes. **a** Bone marrow–derived lymphoid progenitor cells migrate to the thymus, where they differentiate into CD3⁺CD4⁺CD8⁻ SP lymphocytes. A subset of SP cells that recognize self-antigens further differentiates into tTregs under the influence of multiple signaling cues. The hypomethylated state of the CNS2 region, also known as the TSDR, is crucial for stabilizing Foxp3 expression and tTreg lineage commitment. **b** In the periphery, CD4⁺ naïve T cells enter the circulation and, upon encountering APCs in mucosal tissues such as the gut, are induced into pTregs in the presence of cytokines, including IL-2 and TGF-β. TGF-β signaling promotes Smad3 binding to the CNS1 region of the Foxp3 locus, a process essential for pTreg differentiation. This figure was created with Biorender.com

tTregs are generated in the thymus from bone marrow–derived progenitor cells, which first differentiate into CD3⁺CD4⁻CD8⁻ double-negative lymphocytes, then progress to CD4⁺CD8⁺ double-positive thymocytes, which subsequently develop into CD4 single-positive cells. Among them, a subset of CD4⁺ SP cells that recognize self-antigens ultimately mature into tTregs (Fig. 1a).^21,22^ The development of tTregs critically depends on TCR recognition of self-antigens presented by major histocompatibility complex (MHC) class II molecules, together with costimulatory signals mediated by CD80/CD86–CD28 interactions and IL-2 signaling.^22^ Additionally, the *Foxp3* locus harbors several regulatory elements, including conserved noncoding sequences (CNSs) 0–3 and the *Foxp3* promoter elements.^9^ Among these, the CNS2 element, which contains a Treg-specific demethylated CpG region (TSDR), is particularly important for establishing stable, heritable Foxp3 expression in tTregs.^23–25^ The demethylation of CNS2 allows the recruitment of multiple transcription factors critical for tTreg differentiation, phenotypic stability, and function, including STAT5, NFAT, RUNX1/CBFB, CREB, and FOXP3 itself.^9^ As TCRs primarily recognize MHC-II molecules carrying self-antigens during development, tTregs are particularly crucial for preventing autoimmunity.^17^

pTregs are generated in the periphery from Foxp3⁻CD4⁺ T cells under tolerogenic conditions (Fig. 1b). Stimulation by environmental antigens derived from microbial and dietary sources, together with signaling molecules such as TGF-β, IL-2, and retinoic acid (RA), promotes their differentiation into pTregs.^20^ In this process, antigen-presenting cells (APCs), especially CD103⁺ dendritic cells (DCs), capture antigens and present them to CD4⁺Foxp3⁻ T cells while secreting TGF-β and RA, thereby driving their differentiation into pTregs.^26^ pTregs reside preferentially in the intestine due to the TGF-β–rich microenvironment. TGF-β promotes Smad3 binding to the noncoding TGF-β/Smad response element CNS1 at the *Foxp3* gene locus, thereby increasing Foxp3 expression and playing a crucial role in pTreg differentiation.^20,23,27^ Notably, short-chain fatty acids (SCFAs) derived from the gut microbiota also facilitate pTreg generation.^28^ Critically, pTregs possess a TCR repertoire that targets exogenous antigens, which is crucial for maintaining immune tolerance and homeostasis at mucosal surfaces exposed to the external environment.^29^

While Foxp3 expression in mice is largely restricted to Tregs, human naïve CD4⁺ T cells can express FOXP3 upon TCR activation.^30,31^ This results in significant heterogeneity among human FOXP3⁺ T cells, comprising both suppressive and nonsuppressive subsets. To better understand the role of FOXP3⁺ T cells in regulating immune responses, human FOXP3⁺CD4⁺ T cells are classified into three major subsets on the basis of the expression levels of FOXP3, CD25, and CD45RA (a marker of naïve T cells):

(i) FOXP3^lo^CD45RA⁺CD25^lo^ cells (Fr. I), representing naïve or resting Tregs;

(ii) FOXP3^hi^CD45RA⁻CD25^hi^ cells (Fr. II), referred to as effector or activated Tregs, which differentiate from Fr. I upon antigen stimulation, express high levels of cytotoxic T-lymphocyte antigen 4 (CTLA-4), and exhibit strong suppressive activity;

(iii) FOXP3^lo^CD45RA⁻CD25^lo^ cells (Fr. III), a mixed population of Tregs and non-Tregs that lack suppressive function and produce proinflammatory cytokines.^31^

This subset classification is essential for assessing immune status and enabling precise therapeutic interventions. By selectively targeting specific FOXP3⁺ Treg populations, it is possible to modulate immune responses, either by suppressing or enhancing them, to address different disease contexts effectively.

### Functional mechanisms of Tregs

**Fig. 2:**
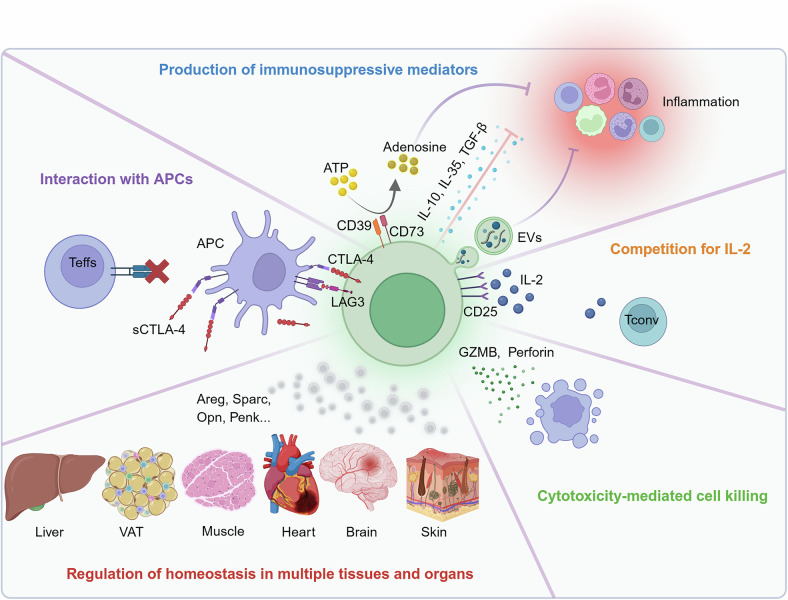
Molecular basis of Treg function. Tregs exert their functions through multiple mechanisms, including (1) expressing CTLA-4 and LAG3 to interfere with DC-mediated T-cell activation; (2) expressing the extracellular enzymes CD39/CD73 to convert ATP and ADP into adenosine, secreting immunosuppressive cytokines such as IL-10, TGF-β, and IL-35, and releasing EVs containing various immunosuppressive molecules; (3) expressing high levels of CD25 to compete for IL-2 in the microenvironment; (4) releasing GZMB and perforin to directly kill target cells; and (5) expressing tissue-specific molecules such as Areg in skeletal muscle, Sparc in the heart, Opn in the brain, and Penk in the skin, among others, to regulate tissue homeostasis and promote post-injury repair. This figure was created with Biorender.com

Tregs exert immunoregulatory functions through direct and indirect mechanisms (Fig. 2). They can interact with the costimulatory molecules CD80/86 and MHC-II on APCs through CTLA-4 and lymphocyte activation gene-3 (LAG3), respectively, thereby disrupting DC-mediated T-cell activation.^32–35^ In addition to direct contact, Tregs secrete soluble CTLA-4 (sCTLA-4), which can remotely target CD80/CD86 on APCs and suppress the differentiation of T helper 1 (Th1) cells.^36^ Tregs highly express the extracellular enzymes CD39 and CD73, which catalyze the conversion of extracellular adenosine triphosphate (ATP) and adenosine diphosphate (ADP) into adenosine. This adenosine binds to adenosine receptor 2A (A2AR) and suppresses effector T-cell (Teff) responses.^37^ Tregs are capable of secreting immune-suppressive cytokines, including IL-10, TGF-β, and IL-35, which suppress the function of proinflammatory cells, such as Th1 cells, Th17 cells and M1 macrophages.^38–40^ Tregs also secrete extracellular vesicles (EVs) containing various microRNAs (such as miR-155 and Let-7d), as well as immunosuppressive molecules (including IL-35, CD73, and Nrp1). These EVs suppress the proliferation and function of inflammatory cells and can induce apoptosis.^41–43^ Moreover, Tregs express high CD25 (IL-2RA) levels, which compete for IL-2 with other cells in the microenvironment, limiting the proliferation and activation of IL-2-sensitive cells, including naïve and effector CD4^+^ T cells.^44,45^ Furthermore, Tregs can mediate cytotoxicity by releasing granzyme B (GZMB) and perforin to lyse Teffs, B cells, and DCs.^46,47^

In addition to their canonical roles in immune regulation, Tregs exhibit specialized functions tailored to specific tissues (Fig. 2). In liver and adipose tissue, Tregs regulate local and systemic metabolic balance, including lipid homeostasis and energy metabolism, while also modulating adipocyte function and adipose tissue inflammation.^5,48,49^ In the contexts of skeletal muscle injury, myocardial infarction, and stroke, Tregs secrete molecules such as amphiregulin (Areg), secreted protein acidic and rich in cysteine (Sparc), and osteopontin (Opn) to promote tissue repair and regeneration.^50–52^ In the skin, Tregs promote the regeneration of hair follicle stem cells and the epithelial barrier through jagged1 (Jag1)-Notch signaling and elevated the expression of preproenkephalin (Penk).^53,54^

### Instability and plasticity of Tregs

The stability of Tregs is critical for their immunoregulatory functions, yet they exhibit dynamic behavior under specific conditions. A study in which the yellow fluorescent protein reporter gene for Foxp3 expression was used revealed that some Tregs that had previously expressed Foxp3 lost its expression, suggesting that Tregs may exhibit a certain degree of instability or plasticity under certain conditions.^55^ Sustained and stable expression of Foxp3 is crucial for Treg stability.^11^ Research has indicated that Foxp3 ubiquitination and degradation regulated by the E3 ligase STIP1 homology and U-box-containing protein 1 (STUB1) promote the conversion of Tregs to the Th1 phenotype, whereas Stub1 inhibition enhances Treg immunosuppressive activity.^56^ Foxp3 expression and Treg stability are governed by multiple molecular mechanisms, including transcription, translation, epigenetic modifications, and posttranslational regulation. For example, transcription factors such as signal transducer and activator of transcription 5 (STAT5), nuclear factor of activated T cells (NFAT), and forkhead box O1 (FOXO1) directly bind to the *Foxp3* promoter and enhancer and modulate its expression.^57–59^ Hypermethylation of the CpG motifs in the CNS regions of the *Foxp3* gene locus negatively affects Foxp3 expression and stability.^9^ Histone acetyltransferases (HATs) and histone deacetylases (HDACs) further regulate *Foxp3* chromatin accessibility by adjusting the acetylation levels of histone lysine residues.^60^ Furthermore, a range of E3 ubiquitin ligases, including STUB1, 70-kDa heat shock proteins (HSP70s), and TNF receptor-associated factor 6 (TRAF6), as well as deubiquitinases such as ubiquitin-specific protease (USP)7, USP22, and USP47, regulate Treg stability and function by modulating the ubiquitination of Foxp3.^56,61–64^ In addition to intrinsic factors, the impact of the microenvironment on Treg stability has garnered increasing attention. Cytokines in the microenvironment (such as IL-2, IL-6, and interferon-gamma (IFN-γ)), along with metabolic products (such as glucose, lactate, and multiple amino acids), and antigenic components play significant roles in regulating Treg stability.^59,65–70^ Recent research has focused on how the metabolic reprogramming of Tregs influences their stability. For example, while appropriate activation of the mammalian target of rapamycin (mTOR) pathway promotes Treg function, excessive mTOR signaling and increased glycolysis can reduce Foxp3 expression and impair Treg suppressive activity.^70–72^ Moreover, under certain conditions, such as hypoxia or IFN-γ stimulation, Tregs can exhibit a state of “fragility”.^73,74^ This state is distinct from conventional Treg instability and is characterized by sustained Foxp3 expression alongside the upregulation of T-bet and IFN-γ, accompanied by impaired immunosuppressive function. These cells are therefore referred to as “fragile Tregs.”^74^

Importantly, the expression of transcription factors associated with other T-cell subsets (such as T-bet for Th1 or RORγt and Stat3 for Th17) by Tregs does not necessarily indicate a loss of immunosuppressive function. In contrast, the expression of T-bet or RORγt may increase the ability of Tregs to localize to specific inflammatory environments and selectively suppress Th1- or Th17-mediated immune responses.^75–77^ This phenomenon fully reflects the complexity of Treg functional regulation, including its context dependence and phenotypic plasticity.

Treg instability and plasticity significantly affect disease progression and the efficacy of immunotherapy. Given the divergent roles of Tregs across disease contexts, it is essential to adopt tailored strategies that either promote Treg instability or maintain Treg stability accordingly. Understanding the intrinsic cellular mechanisms and external environmental factors that affect the stability of Tregs across different diseases can facilitate the development of more precise Treg-targeted immunotherapies with significant clinical value.

## Tregs in cancer

Cancer remains one of the leading causes of death worldwide, and its onset is often accompanied by immune system activation.^78^ The immune system plays a dual role in cancer: it can eliminate cancer cells or inhibit their growth, thereby suppressing tumor progression; however, it can also foster a tumor-promoting microenvironment that facilitates cancer development.^78,79^ As key regulators of immune responses, Tregs play critical roles in cancer initiation, progression, and therapeutic outcomes.^7^

### Treg accumulation in the TME

**Fig. 3:**
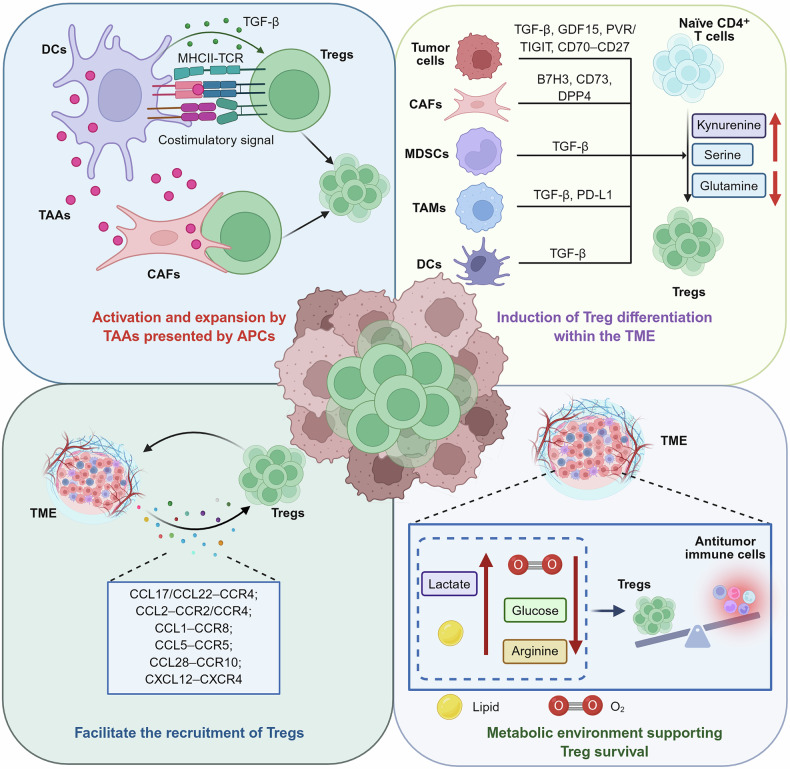
Mechanisms underlying Treg accumulation in the TME. Several mechanisms contribute to the preferential accumulation of Tregs in the TME. (1) TAAs presented by APCs, such as DCs and CAFs, are recognized by Tregs via TCR-mediated recognition of MHC-II–peptide complexes, thereby promoting Treg activation and proliferation. (2) Within the TME, various stromal and immune cells support Treg differentiation through direct interactions and the secretion of soluble factors. Additionally, metabolic cues within the TME can further enhance the induction of Tregs. (3) The recruitment of Tregs is facilitated by several chemokine–receptor axes that guide Tregs into the TME. (4) The metabolic landscape of the TME favors Treg survival and functional dominance. This figure was created with Biorender.com

Tumor-infiltrating Tregs (TI-Tregs) may originate from tTregs or from the conversion of naïve CD4^+^ T cells. Although some studies have suggested that tTregs may represent a predominant source, the exact origin remains uncertain and may vary depending on the tumor type.^80–82^ Within the TME, Tregs often constitute a markedly greater proportion of total T cells than do those in peripheral blood. In some tumor types, such as colorectal and gastric cancers, they can reach or even exceeding 50% of the intratumoral CD4⁺ T-cell population.^83,84^ Multiple factors contribute to the accumulation and functional shaping of Tregs in the TME, including their activation and expansion by tumor-associated antigens (TAAs) presented by APCs; the induction of Treg differentiation within the TME through various mechanisms; the recruitment of Tregs via chemokines secreted by multiple cell types within the TME; and the metabolic characteristics of the TME that support Treg viability and immunosuppressive function (Fig. 3).

The TME is enriched with TAAs, which are largely self-antigens. The TCR repertoire of tTregs is biased toward recognizing self-antigens, allowing them to clonally expand within the TME.^83^ Myeloid cells, particularly DCs, are the primary APCs with complex biological characteristics.^85^ They can cross-present TAAs to T cells via MHC molecules, thereby participating in the induction of immune responses. The DC population is heterogeneous and comprises conventional DCs (cDCs, including cDC1s and cDC2s), plasmacytoid DCs (pDCs), and monocyte-derived DCs (moDCs).^85,86^ Recently, single-cell technologies have identified a distinct and highly conserved subset of activated DCs, known as mature regulatory DCs (mregDCs, also termed DC3s or LAMP3⁺ DCs), in various cancers.^87^ In the TME, after acquiring TAAs, DCs migrate to the tumor-draining lymph nodes (TDLNs), where they induce the activation and expansion of Tregs, which subsequently migrate from the TDLNs to the tumor tissue. Furthermore, DCs can directly activate Tregs within the TME.^86,88^ Additionally, Tregs can receive TGF-β and costimulatory signals from DCs, further promoting their proliferation.^89,90^ Single-cell analysis revealed that DCs interact with Tregs via pathways such as the MHC II–TCR, ICOSLG–ICOS, IL-18–IL18R1, and IL-1–IL1R1 signaling pathways. These interactions not only enhance Treg clonal expansion but also strengthen their immunosuppressive capacity.^90^ Recent studies have emphasized the role of mregDCs in recruiting, activating, and promoting the expansion of Tregs within tumors, thereby triggering robust immune suppression.^91–93^ Interaction with mregDCs prompts Tregs to upregulate costimulatory and inhibitory molecules on their surface, which further enhances their interactions with mregDCs and suppresses the migration of mregDCs to TDLNs. This migration blockade limits the ability of mregDCs to present antigens to tumor-specific Teffs, thereby hindering antitumor immune responses.^93^ In addition to DCs, α-SMA⁺ cancer-associated fibroblasts (CAFs) can phagocytize tumor antigens and form immune synapses with Tregs within the TME, promoting Treg activation and proliferation in an antigen-specific manner.^94^ Finally, in patients with melanoma, certain tumor cells express MHC-II molecules, enabling the direct activation and expansion of tumor neoantigen-specific Tregs.^95^

In the TME, various cell types, including tumor cells, APCs, CAFs, and myeloid-derived suppressor cells (MDSCs), drive Treg differentiation through multiple mechanisms. For example, the TME is enriched with TGF-β, which potently induces Treg differentiation and accumulation.^82,96,97^ Additionally, growth differentiation factor 15 (GDF15), which belongs to the TGF-β family, has been shown to enhance Treg induction in hepatocellular carcinoma (HCC).^98^ In head and neck squamous cell carcinoma (HNSCC), cancer cells expressing Plac1 recruit CD4⁺ T cells via the CXCL11/CXCR3 axis and subsequently induce their differentiation into Tregs through the PVR/TIGIT pathway.^99^ In patients with nasopharyngeal carcinoma, cancer cells reprogram lipid metabolism through CD70‒CD27 interactions, polarizing naïve CD4⁺ T cells toward a Treg phenotype.^100^ Moreover, breast cancer cell-derived GM-CSF upregulates aryl hydrocarbon receptor (AHR) levels in macrophages, leading to increased PD-L1 expression and promoting the differentiation of Tregs.^101^ CAFs are recognized as key contributors to tumor progression, among which the CAF-S1 subset plays a prominent role. In triple-negative breast cancer (TNBC), CAF-S1 promotes T-cell accumulation via CXCL12 secretion and drives their differentiation into Tregs through B7H3, CD73, and DPP4.^102^ Metabolic factors within the TME also critically contribute to Treg induction. For example, kynurenine (Kyn) facilitates Treg differentiation through activation of the transcriptional regulator AHR.^103^ Serine and palmitic acid accumulation enhance sphingolipid biosynthesis in Tregs, and the resulting metabolite sphingosine promotes Treg differentiation by directly interacting with the transcription factor c-Fos.^104^ Cancer cells consume large quantities of glutamine to fulfill their biosynthetic and energetic needs, resulting in a glutamine-deprived environment.^105^ Under such conditions, the activation of naïve CD4⁺ T cells preferentially drives their differentiation into Tregs.^106^ A glutamine-deprived environment also induces macrophages to secrete IL-23, which further promotes Treg proliferation and the expression of effector molecules.^107^

Tregs can also be recruited into the TME through chemotaxis (Table 1). Tumor cells, CAFs, DCs, and tumor-associated macrophages (TAMs) secrete various chemokines to recruit Tregs into the TME through specific chemotactic axes, including CCL17/CCL22–CCR4,^108^ CCL2–CCR2/CCR4,^109,110^ CCL1–CCR8,^111,112^ CCL5–CCR5,^113,114^ CCL28–CCR10,^115^ and CXCL12–CXCR4.^116,117^ The predominant chemokine axes differ across cancer types, and targeting Treg chemotaxis represents potential therapeutic strategies to reduce their intratumoral accumulation.

**Table 1:** Chemokines mediating Treg recruitment in the TME

Chemokine ligand	Ligand source	Receptor expression on Tregs	Tumor type	Reference
CCL17	Cancer cell, TAM, TAN, CAF, DC	CCR4	HCC, breast cancer, lymphoma, GC, melanoma, CRC, NSCLC	^108,454,514–516^
CCL22	Cancer cell, TAM, DC, CAF, TAN	CCR4	Breast cancer, HCC, ovarian carcinoma, melanoma, Lung cancer, GC, CRC, cervical cancer	^108,201,291,292,517–519^
CCL2	Cancer cell, TAN, TAM, microglia	CCR2/CCR4	Glioblastoma, retinoblastoma, HCC	^109,110,454,520^
CCL1	Fibroblast, cancer cells, T cell	CCR8	Breast cancer	^111,521^
CCL5	Cancer cell	CCR5	pancreatic cancer, colon cancer	^113,114^
CCL28	Cancer cell, epithelial cells	CCR10	Ovarian cancer, CRC	^115,522^
CXCL12	CAF, cancer cell	CXCR4	Breast Cancer, OSCC, RCC, ovarian cancer, BCL	^116,117,523–526^

*TAM* tumor-associated macrophage, *TAN* tumor-associated neutrophil, CAF cancer-associated fibroblast, *DC* dendritic cell, *HCC* hepatocellular carcinoma, *GC* gastric cancer, CRC colorectal cancer, *NSCLC* non-small cell lung cancer, *OSCC* oral squamous cell carcinoma, *RCC* renal cell carcinoma, *BCL* B cell lymphoma

Finally, metabolic alterations within the TME—such as the accumulation of lactate and fatty acids, as well as deficiencies in oxygen, glucose, and arginine—create conditions that favor Treg survival and functional activity.^69,118–120^ This phenomenon will be discussed in greater detail in the following section on Treg stability in tumors.

In summary, these mechanisms enable Tregs to stand out among various immune cells, making them the most representative immunosuppressive cell type within the TME.

### Role of Tregs in the TME

Tregs promote tumor progression primarily by suppressing antitumor immune responses, thereby enabling tumor immune evasion. As described previously, Tregs suppress immune responses through multiple mechanisms. These factors include high expression of molecules such as CTLA-4 and LAG-3 to compete with APCs; the overexpression of CD25 to capture IL-2; the secretion of immunosuppressive cytokines such as IL-10, IL-35, and TGF-β; and the conversion of extracellular ATP/ADP to adenosine via CD39 and CD73.

In addition to these canonical mechanisms, several unique functions of Tregs within the TME have been revealed in recent studies. For example, Tregs can express lymphotoxin (LT) α1β2, which activates LTβ receptors (LTβRs) on tumor cells and lymphatic endothelial cells, triggering noncanonical NF-κB signaling to promote tumor growth and lymphatic metastasis.^121^ Additionally, Tregs express receptor activator of nuclear factor-κB ligand (RANKL), which binds to its receptor RANK on cancer cells to activate inhibitor of nuclear factor-κB kinase-α (IKK-α), thereby promoting tumor metastasis.^122^ Tregs also suppress IFN-γ production by CD8⁺ T cells, thereby indirectly supporting fatty acid synthesis, mitochondrial integrity, and the survival of M2-like TAMs through SREBP1 signaling, which further contributes to immune evasion.^123^ Activated Tregs express the TGF-β activator LRRC32 (also known as GARP) and integrin αvβ8, enabling the activation of latent TGF-β within the TME and leading to the suppression of other immune cells.^124,125^ Furthermore, in various cancer models, Tregs upregulate CXCR3, which allows them to interact with CXCL9^+^ type 1 cDC1s, impairing tumor antigen cross-presentation to CD8^+^ T cells and restricting antitumor immunity.^126^ Reactive oxygen species (ROS) within the TME can induce Treg apoptosis; notably, these apoptotic Tregs exhibit increased immunosuppressive activity via the release of large amounts of ATP, which is subsequently converted into immunosuppressive adenosine via CD39 and CD73.^127^ This mechanism may represent a major mode of Treg-mediated immunosuppression in tumors and contribute to poor responsiveness to anti–programmed death-ligand 1 (PD-L1)/programmed cell death protein 1 (PD-1) therapies in patients with cancer.^127^ Tissue-resident Tregs are characterized by high expression of ST2 (IL1RL1), and a subset of ST2⁺ Tregs has been identified in tumors. These cells express high levels of Areg, which induces a profibrotic and immunosuppressive state in CAFs via the AREG/EGFR axis.^128^

However, Tregs do not always contribute to cancer progression. In certain cancers—such as colorectal cancer (CRC), head and neck cancers, and classical Hodgkin lymphoma—higher Treg numbers correlate with better patient prognosis.^7,84^ This paradox may be attributed to the phenotypic and functional heterogeneity of Tregs. CRC can be categorized into two subtypes on the basis of the phenotype of TI-Tregs. In subtype A CRC, tumors are predominantly infiltrated by immunosuppressive FOXP3^hi^ Fr. II T cells, where higher FOXP3⁺ T-cell numbers correlate with a worse prognosis. In contrast, subtype B CRC is enriched with nonsuppressive FOXP3^lo^ Fr. III T cells that exhibit unstable Foxp3 expression and secrete inflammatory cytokines, with a positive correlation between Foxp3⁺ T-cell numbers and patient prognosis.^84^ Moreover, in certain tumors, depletion of Tregs may disrupt the immune balance of the TME and trigger compensatory regulation by other stromal or immune cell types.^129,130^ For example, in a pancreatic cancer model, Treg ablation led to reduced TGF-β levels, which reshaped the fibroblast compartment. This resulted in the loss of tumor-restraining αSMA⁺ fibroblasts and the upregulation of chemokines such as Ccl3, Ccl6, and Ccl8, leading to massive myeloid cell recruitment, which subsequently restored immunosuppression and promoted cancer progression.^129^ In CRC mouse models and patients, the weakened immunosuppressive function of Tregs enhances Th17-type inflammation, which may induce PD-1 expression on CD8⁺ T cells and impair their antitumor activity, thereby paradoxically accelerating CRC initiation and progression.^131^ These findings highlight the importance of precisely evaluating Treg function across cancers for designing effective immunotherapies.

### Stability of Tregs in the TME

Although Tregs exhibit a degree of instability and plasticity, they tend to maintain phenotypic and functional stability within tumors, thereby sustaining their immunosuppressive capacity.^13,132^ Systemic Treg depletion often triggers severe autoimmune diseases, making it an impractical approach. Instead, a promising antitumor immunotherapy strategy focuses on leveraging Treg instability or fragility to promote their functional dysregulation, thus attenuating their suppressive function and enhancing antitumor immune responses.^7,13,132,133^ A thorough understanding of the key factors that maintain the stability of TI-Tregs may provide new opportunities for improving antitumor immunotherapy. While several excellent reviews have addressed the stability of Tregs,^13,133^ a comprehensive summary of how various cues within the TME influence the stability of Treg function and phenotype is still lacking.

#### Impact of metabolism on the stability of Tregs in the TME

**Fig. 4:**
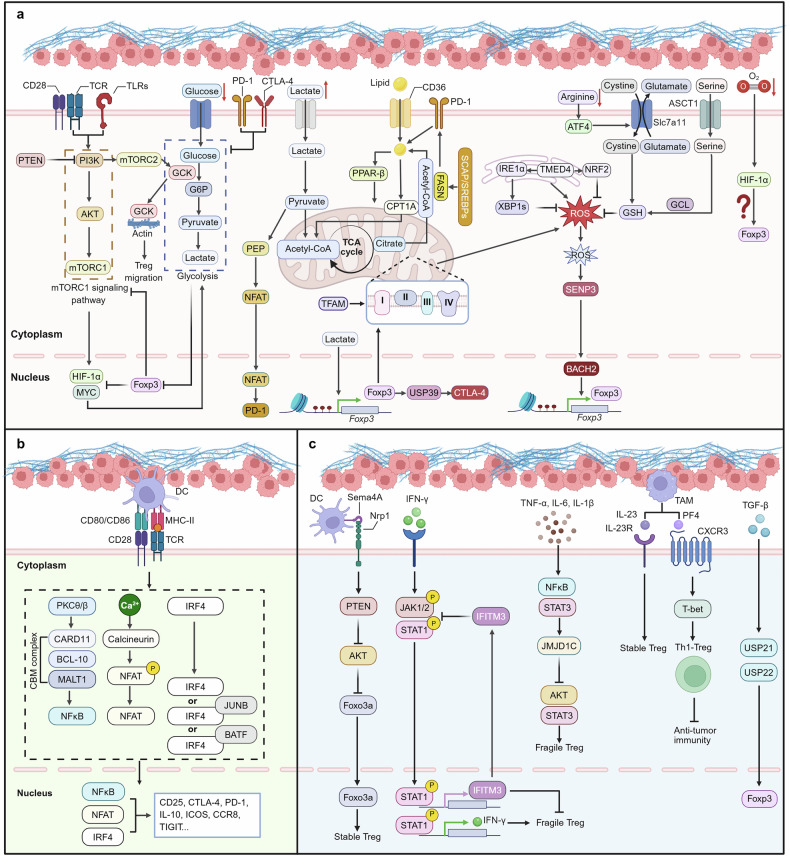
Mechanisms underlying Treg stability in the TME. **a** Excessive glycolysis undermines Treg stability. Tregs limit glycolysis and upstream signals via Foxp3, PTEN, PD-1, and CTLA-4. mTORC2 promotes the expression of GCK to facilitate Treg migration. Foxp3 and TFAM promote mitochondrial ETC complex expression and increase OXPHOS to meet energy demands. Moderate ROS stabilize Tregs via the SENP3–BACH2 axis. To mitigate excessive ROS, Tregs employ TMED4 and GSH to suppress oxidative stress. In the lactate-rich TME, Tregs utilize lactate for energy and stability, with lactate promoting PD-1 and CTLA-4 expression via NFAT and the Foxp3‒USP39 axis. Additionally, Tregs express CD36 and CPT1A to promote fatty acid uptake and FAO while also activating the SCAP/SREBP pathway to sustain FAS and maintain lipid metabolic homeostasis. Moreover, PD-1 enhances lipid metabolism in Tregs. Arginine deficiency induces ATF4 and SLC7A11 expression, facilitating cystine–glutamate exchange and GSH synthesis. Tregs also use serine for GSH production. Hypoxia regulates Tregs via HIF-1α, although the underlying mechanisms remain debated. **b** TCR engagement activates multiple downstream pathways that increase Treg stability. The CBM signaling complex stimulates NF-κB activation; the calcium/calmodulin-dependent phosphatase calcineurin facilitates NFAT nuclear translocation; and TCR signaling promotes IRF4 expression, thereby upregulating molecules associated with Treg function and stability. **c** DC-derived Sema4A binds Nrp1 on Tregs, stabilizing them via PTEN. Tregs counteract IFN-γ–mediated fragility through the STAT1–IFITM3 loop. Inflammatory signals upregulate JMJD1C to support Treg stability. TAM-derived IL-23 and PF4 further enhance Treg function and stability. High TGF-β levels in the TME promote USP21 and USP22, sustaining Foxp3 stability. This figure was created with Biorender.com

Owing to the unique metabolic characteristics of tumor cells, changes in nutrient availability, metabolic byproducts, and oxygen within the TME can influence the presence and function of various immune cells, including TI-Tregs. Within the TME, Tregs also adopt flexible metabolic strategies to address the challenges posed by environmental changes.^105,134^ Next, we discuss the impact of different metabolic pathways and metabolites within the TME on Treg stability (Fig. 4a).

##### Impact of glycolysis and oxidative phosphorylation (OXPHOS) on Treg stability in the TME

In 1924, Otto Warburg first reported that tumor cells preferentially use glycolysis over OXPHOS even under oxygen-rich conditions, a phenomenon known as the “Warburg effect,” to meet their high energy and biosynthetic demands.^135^ This leads tumor cells to compete with Teffs for glucose, which also relies heavily on glycolysis to meet their energy demands.^136^ In contrast to Teffs, Tregs depend primarily on OXPHOS and fatty acid oxidation (FAO) for their metabolic needs, giving them a metabolic advantage in the low-glucose TME.^134^ Tregs maintain their distinct metabolic profile through multiple regulatory mechanisms. The activated phosphoinositide 3-kinase (PI3K)–protein kinase B (Akt)–mTORC1 signaling axis promotes glycolysis via the induction of Myc and the α subunit of hypoxia-inducible factor-1 (HIF-1α).^136^ In Tregs, Foxp3 suppresses the upregulated mTORC1 signaling pathway following TCR and CD28 stimulation and downregulates c-Myc expression, thereby strictly limiting glycolytic activity and promoting a metabolic preference for OXPHOS and FAO.^71,137^ In addition, Tregs suppress glycolysis by upregulating PTEN to inhibit the PI3K signaling pathway and restrict mTORC1 and c-Myc activity through autophagy.^138–140^ The expression of inhibitory receptors such as CTLA-4 and PD-1 on Tregs also contributes to the suppression of glycolysis.^141,142^ In addition to inhibiting glycolysis, Foxp3 directly enhances the expression of components of the electron transport chain (ETC), thereby promoting OXPHOS activity.^143^ Lkb1 and AMPK also contribute to maintaining mitochondrial metabolic homeostasis in Tregs under both homeostatic and disease conditions.^144,145^ Through these mechanisms, Tregs exhibit a metabolic profile distinct from that of other T-cell subsets.

Numerous studies have demonstrated that excessive glycolysis impairs the stability of Tregs and compromises their immunosuppressive function.^71,138,139,146^ Inhibiting tumor cell glycolysis increases glucose availability within the TME, thereby reducing Treg stability and suppressive capacity and enhancing the efficacy of immune checkpoint blockade (ICB) therapy.^68,147,148^ Additionally, Tregs with high glucose affinity in tumors exhibit upregulated expression of glycolysis-related genes, which are associated with impaired suppressive function and reduced stability.^120^ However, some studies have reported that tumor-associated Tregs rely primarily on glucose metabolism, which facilitates immunosuppressive activity by inducing effector T-cell senescence through cross-talk.^149^ Moreover, glycolysis may promote Treg proliferation via activation of the PI(3)K–Akt–mTORC1 pathway and increase Treg migration via PI3K–mTORC2-mediated glucokinase (GCK)–actin binding.^71,150,151^ Consequently, the inhibition of glycolysis in TI-Tregs impairs their migratory capacity and suppressive function, ultimately restraining tumor progression.^149,152^ These seemingly contradictory findings may be attributed to differences in experimental models, tumor types, and therapeutic interventions. Therefore, further in-depth and systematic investigations are urgently needed to elucidate the precise role of glycolysis in TI-Tregs and to assess the feasibility and applicability of targeting this pathway for tumor immunotherapy.

OXPHOS and mitochondrial metabolism are crucial for maintaining Treg stability in tumors. Notably, knockout of mitochondrial transcription factor A (TFAM), which is crucial for mitochondrial OXPHOS, causes increased methylation in the TSDR of the *Foxp3* gene locus in Tregs, disrupting Foxp3 expression stability and enhancing antitumor immune responses.^153^ Similarly, when Tregs exhibit deficiencies in ETC complexes I or III, they show reduced stability and impaired immunosuppressive function but enhanced antitumor immune responses.^137,154^ AMPK is essential for maintaining normal mitochondrial function in TI-Tregs. AMPK deficiency leads to the accumulation of the DNMT1 protein, resulting in hypermethylation at the promoters of key metabolic genes, which impairs mitochondrial metabolism and Treg suppressive function, ultimately delaying tumor progression.^144^ In addition to directly affecting Treg stability and function, ROS, the byproducts of mitochondrial respiration, are significant in regulating Treg stability. The impact of ROS on Tregs remains controversial: moderate ROS accumulation seems to favor Treg stability, whereas excessive ROS impairs Treg-mediated immunosuppressive functions.^155–157^ After TCR and CD28 stimulation, Sentrin/SUMO-specific protease 3 rapidly accumulates in Tregs in an ROS-dependent manner, positively regulating Treg stability and function by promoting BTB and CNC homolog 2 (BACH2) desumoylation.^155^ To prevent excessive intracellular ROS accumulation, Tregs also upregulate transmembrane emp24 domain-containing protein 4 (TMED4), which promotes ROS homeostasis and maintains Foxp3 expression and suppressive capacity by activating the IRE1α–XBP1 signaling pathway and NRF2-mediated antioxidant responses.^158^ In addition, Tregs synthesize glutathione (GSH) to scavenge excessive ROS.^156^

##### Impact of lipid metabolism on Treg stability in the TME

Tregs utilize fatty acid-binding protein (FABP), CD36, and carnitine palmitoyltransferase 1A (CPT1A) to take up free fatty acids and transfer them to the mitochondria for lipid metabolism.^119,159^ Within the TME, Tregs upregulate CD36 expression to support mitochondrial fitness via a peroxisome proliferator-activated receptor (PPAR)-β–dependent mechanism. CD36 knockout in Tregs weakens the abundance and suppressive activity of TI-Tregs, inhibits tumor growth, and enhances the efficacy of ICB therapy without causing severe autoimmunity.^119^ Similarly, inhibition of CPT1A impairs FAO, diminishes the immunosuppressive capacity of Tregs, and improves survival in murine models of glioma.^152^ In addition, lipid peroxidation can lead to ferroptosis. Tregs maintain homeostasis within the TME by expressing glutathione peroxidase 4 (Gpx4), which prevents lipid peroxidation and ferroptotic cell death.^160^ In addition to FAO, fatty acid synthesis (FAS) is critical for maintaining Treg stability in tumors. Tregs activate FAS through sterol regulatory element-binding proteins (SREBPs), which are regulated by SREBP cleavage-activating proteins. This process promotes de novo lipid synthesis and increases PD-1 expression, thereby preventing Tregs from acquiring an unstable and fragile phenotype.^161^ Similarly, PD-1 reportedly promotes Treg stability in tumors by enhancing fatty acid metabolism and OXPHOS.^162^ In tumors, Tregs also take up glucose for lipid synthesis, which promotes their proliferation, stability, and immunosuppressive function. Inhibition of lipid synthesis via an acetyl-CoA carboxylase inhibitor induces regression of HCC in mouse models.^150^

##### Impact of lactate on Treg stability in the TME

Highly glycolytic tumors produce abundant lactate, which drives the metabolic reprogramming of Tregs.^105^ Unlike Teffs, Tregs can take up lactate through monocarboxylate transporter-1 (MCT1) and convert it into pyruvate for mitochondrial OXPHOS via lactate dehydrogenase.^120,137^ MCT1-deficient Tregs can maintain immune tolerance in peripheral tissues but struggle in the TME, where impaired lactate utilization forces Tregs to upregulate glycolysis, leading to IFN-γ expression and an unstable, less suppressive phenotype.^120^ In addition, lactate enhances mitochondrial function by promoting MGAT1 transcription and its translocation into mitochondria, which, in turn, strengthens Treg proliferation and suppressive function.^163^ In addition to improving metabolism, Tregs use MCT1 to take up lactate and metabolize it into phosphoenolpyruvate (PEP). PEP acts as a metabolic checkpoint by increasing cytosolic Ca²⁺ levels, thereby promoting the nuclear translocation of NFAT1 and upregulating PD-1 expression in Tregs.^164^ Furthermore, lactate also modulates the expression of another immune checkpoint molecule, CTLA-4, on Tregs. Lactate intake promotes USP39-mediated RNA splicing, which enhances CTLA-4 expression via Foxp3, maintaining the phenotype and functional state of TI-Tregs.^165^ Lactate also stabilizes Treg function by promoting myosin lactylation, enhancing TGF-β signaling, and suppressive function.^67^ Using lactate dehydrogenase inhibitors to reduce lactate levels can delay tumor growth, decrease the number of Tregs in tumors, impair the suppressive functions of Tregs, and synergize with various ICB therapies.^67,164^

##### Impact of amino acid metabolism on Treg stability in the TME

Similarly, amino acid metabolism in Tregs within the TME significantly affects their stability. Tumor cells express indoleamine and tryptophan 2,3-dioxygenases (IDO/TDO), which convert tryptophan to kynurenine.^103^ Kynurenine activates AHR, leading to Treg maturation and the expression of immunosuppressive factors, thereby facilitating tumor immune evasion.^103^ The use of AHR inhibitors can delay the progression of IDO- and TDO-expressing tumors and promote antitumor T-cell immunity.^103,166^ Moreover, the TME is characterized by arginine deficiency.^69^ Arginine deprivation induces ATF4 expression in T cells, which upregulates SLC7A11 expression. SLC7A11 mediates the exchange of extracellular cystine and intracellular glutamate, thereby promoting glutathione (GSH) synthesis and maintaining redox homeostasis. This compensatory pathway mitigates the accumulation of ROS, thereby maintaining Foxp3 expression and Treg-associated functions.^69^ Although anti-VEGF therapy inhibits angiogenesis, it fails to improve overall survival in patients with glioblastoma.^167^ Subsequent studies revealed that VEGF blockade elevates glutamate levels within the TME, which promotes Treg proliferation, activation, and suppressive function.^168^ Combining anti-VEGF antibodies with CD25-blocking agents to deplete Tregs can overcome this immunosuppressive effect.^168^ Tregs utilize serine to synthesize GSH via glutamate-cysteine ligase (GCL) and glutathione synthetase, thereby scavenging excessive ROS and ensuring their stability and survival within the TME. Tregs with GCL deficiency exhibit increased serine uptake and de novo synthesis, which enhances mTOR activity while reducing Foxp3 expression, ultimately resulting in severe autoimmunity and enhanced antitumor immunity.^156^

##### Impact of hypoxia and HIF-1α signaling on Treg stability in the TME

Hypoxia within the TME induces the upregulation of HIF-1α in Tregs; however, its impact on Treg stability remains controversial. High HIF-1α levels can impair Treg suppressive function by promoting glycolysis and binding to Foxp3, triggering its degradation.^169,170^ Conversely, some studies have suggested that HIF-1α interacts with the Foxp3 promoter in Tregs, increasing Foxp3 transcription.^171^ Studies in tumors have also shown conflicting results. For example, in Foxp3-specific HIF-2α-knockout mice, tumor growth was slowed, potentially owing to the elevation of HIF-1α after HIF-2α deletion, resulting in impaired Treg immunosuppressive function.^172^ However, within the TME, HIF-1α was also found to increase the expression of USP22, which maintains Foxp3 expression and immunosuppressive function through deubiquitination.^61,173^ In addition to affecting Treg stability, HIF-1α was found to support the intratumoral migration of Tregs through glycolysis; however, its immunosuppressive function was impaired.^152^ These contradictory findings may be related to the differing levels of HIF-1α elevation in Tregs across different TMEs. Further investigation is needed to clarify the effects of HIF-1α on Tregs in different tumor types and the mechanisms driving these differences to optimize Treg-targeted cancer immunotherapies.

#### Impact of TCR signaling on Treg stability in the TME

Like other T cells, Tregs require TCR signaling. The TME is rich in antigens presented by APCs, which engage Tregs through their TCRs (Fig. 4b).^83^ This signaling is critical for Treg development, activation, stability, and execution of their immunosuppressive functions.^174,175^ Upon TCR and CD28 costimulation, protein kinase Cθ is activated, which directly phosphorylates the caspase recruitment domain-containing protein 11 (CARD11) linker region, promoting the CARD11–BCL-10–MALT1 (CBM) signaling complex, which is crucial for activating nuclear factor-kappa B (NF-κB) and mitogen-activated protein kinase (MAPK) signaling cascades.^176^ These pathways are essential for Treg development and functional stability.^176,177^ Mice with CBM deficiencies induced by pharmacological inhibition or genetic editing exhibit severe defects in Tregs, which can induce an unstable Treg phenotype in the tumor, supporting antitumor immunity.^178^ Additionally, the activation of the CBM complex and MALT1 promotes CTLA-4 expression, suggesting that MALT1 inhibition may have a synergistic effect with ICB therapies.^179^ In addition, TCR activation influences Treg stability and function through NFAT. Upon TCR activation, intracellular calcium levels increase, activating the calcium/calmodulin-dependent phosphatase calcineurin, which dephosphorylates NFAT and promotes its translocation into the nucleus to exert regulatory functions.^180^ Within the nucleus, NFAT forms a complex with Foxp3, promoting the expression of markers and effector molecules such as IL2RA and CTLA-4.^181^ NFAT cooperates with Smad to activate *Foxp3* enhancers, and it binds to CNS2 to facilitate its interaction with the *Foxp3* promoter; both mechanisms are critical for the induction and stabilization of Foxp3 expression.^24,58^ TCR stimulation also increases the expression of IRF4, a transcription factor critical for maintaining Treg stability.^182^ IRF4 cooperates with the activator protein-1 (AP-1) family members BATF and JUNB to promote the expression of molecules associated with Treg function, such as ICOS, CCR8, TIGIT, and CTLA-4.^183,184^ IRF4 deficiency in Tregs compromises their suppressive function and promotes tumor immunity.^183^ Notably, a scRNA-seq and TCR-seq study revealed that nearly all TAA-specific Tregs exhibited a Th1-like phenotype within 2 weeks of entering the TME, characterized by downregulation of Foxp3 and marked upregulation of T-bet and IFN-γ.^185^ This phenomenon may be attributed to the fact that TAAs are largely self-antigens and that self-antigen-specific Tregs are more prone to differentiate into Th1-like Tregs.^81,186^

#### Impact of proteins and cytokines on Treg stability in the TME

Within the TME, various extracellular proteins and cytokines can regulate the stability of Treg function and phenotype (Fig. 4c). In tumor-bearing mice, approximately 90% of tumor-infiltrating Tregs express Nrp1. After binding with semaphorin 4A (Sema4A) in the TME, Nrp1 mediates PTEN recruitment, inhibits Akt activity, and stabilizes TI-Tregs, which helps maintain their immunosuppressive function within tumors.^187^ In the TME, Nrp1-deficient Tregs display a fragile phenotype and produce elevated levels of IFN-γ, which in turn induces fragility in neighboring Nrp1-expressing Tregs.^74^ This IFN-γ-mediated Treg fragility is essential for the therapeutic efficacy of anti-PD-1 treatment.^74^ Tregs can also counteract IFN-γ-induced fragility through the STAT1–IFITM3 feedback loop. IFN-γ induces STAT1 phosphorylation, and phosphorylated STAT1 translocates into the nucleus to promote the expression of IFITM3.^188^ IFITM3, in turn, inhibits STAT1 phosphorylation and promotes its degradation, thereby blocking sustained activation of the IFN-γ signaling pathway and maintaining Treg function and stability.^188^ IL-33 also contributes to the maintenance of Treg stability and is associated with the progression of various malignancies. IL-33 deficiency leads to the downregulation of Nrp1 expression and the activation of mTOR in Tregs while promoting the production of IFN-γ and the emergence of a fragile phenotype through the NF-κB–T-bet axis.^189^ In addition, tumor-associated cytokines such as TNF, IL-6, and IL-1β can induce the expression of the histone demethylase JMJD1C via the NF-κB and STAT3 signaling pathways, which suppress Akt and STAT3 signaling and prevents Tregs from acquiring a fragile phenotype.^66^ TAMs secrete IL-23, which stabilizes Treg function and Foxp3 expression through the IL-23/IL-23R axis, enhancing immunosuppression and impairing antitumor immunity.^190^ TAMs can also secrete platelet factor 4 (PF4), which binds to CXCR4 to promote the polarization of Tregs into Th1-Tregs. Although these Tregs also express T-bet, they exhibit stronger immunosuppressive capacity.^77^ In addition, tumor-derived TGF-β specifically promotes the expression of Usp22 and Usp21 in Tregs, promoting Foxp3 stability.^61^ Moreover, GDF15 interacts with CD48 and blocks Foxp3 ubiquitination-mediated degradation by downregulating STUB1 via suppression of the extracellular signal-regulated kinase (ERK)/AP-1 pathway, enhancing the immunosuppressive function and stability of Tregs.^98^

Through the above mechanisms, Tregs can maintain the stability of their function and phenotype within the TME, exerting immunosuppressive effects and contributing to the formation of a milieu that supports tumor progression. Furthermore, these mechanisms offer new potential targets for cancer immunotherapy. Although current studies have revealed some of these regulatory pathways, further in-depth investigations are needed to elucidate the key molecular mechanisms and dynamic changes underlying Treg stability within the TME, thereby providing a more solid theoretical foundation for Treg-targeted antitumor immunotherapeutic strategies.

### Tregs in conventional tumor therapies

**Fig. 5:**
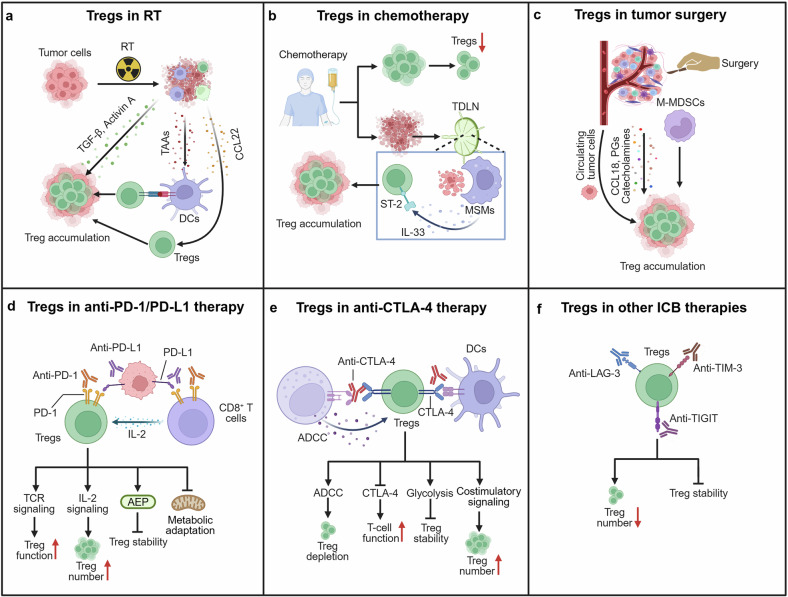
Tregs respond to multiple cancer therapies and influence treatment efficacy. **a** RT promotes Treg accumulation in the TME via increased TGF-β and Activin A, enhances Treg recruitment, and induces Treg expansion through antigen release from dying tumor cells. **b** Chemotherapy agents reduce Treg numbers, whereas necrotic tumor fragments in TDLNs stimulate MSMs to produce IL-33, promoting Treg recruitment. **c** Surgical trauma, along with the release of CCL18, PGs, and catecholamines, enhances Treg proliferation, and surgery-induced expansion of M-MDSCs further supports Treg function and expansion. **d** Anti-PD-1 therapy may promote Treg expansion via enhanced TCR signaling or increased IL-2, or induce instability via metabolic stress or AEP upregulation. **e** Anti-CTLA-4 therapy reduces Tregs through Fc-dependent ADCC, impairs CD80/CD86 competition, and promotes glycolysis-associated instability but may also increase CD28 costimulation and Treg proliferation. **f** Targeting LAG-3, TIM-3, and TIGIT may reduce Treg numbers and impair Treg stability and function. This figure was created with Biorender.com

Conventional cancer treatments include radiotherapy (RT), chemotherapy, and surgery. Increasing evidence has suggested that these treatments can remodel the TME, thereby influencing therapeutic outcomes.^191^ As crucial components of the TME, Tregs play a significant role in this process (Fig. 5).

#### Tregs in RT

The impact of RT on the TME is multifaceted. First, radiation-induced tumor cell death leads to the release of immunogenic tumor antigens, promoting immune cell activation.^192,193^ Second, radiation alters the metabolic landscape of tumor cells and the TME, thereby influencing the composition and function of various immune cells within the TME.^194^ In addition, radiation stimulates tumor cells to release various mediators, which modulate both the quantity and function of immune cells.^193^ Moreover, the complexity of RT-induced immune modulation is further shaped by factors such as the radiation regimen and tumor type.^195^ Tregs undergo dynamic changes in response to RT, which can significantly influence therapeutic outcomes (Fig. 5a).

Most studies have suggested that RT increases the number of TI-Tregs through multiple mechanisms. In mouse models of breast cancer, RT elevates Treg levels in both tumors and peripheral tissues, thereby compromising treatment efficacy. This effect is mediated by increasing the levels of TGF-β and activin A, which increase Treg abundance via the SMAD2/3 signaling pathway.^196^ Another study revealed that in some murine tumor models, post-RT Treg proliferation occurs independently of TGF-β and IL-33 signaling and is driven primarily by local intratumoral expansion.^197^ Additionally, in a TC-1 tumor model, poor tumor control following RT was attributed to increased tumor antigen availability, which activated Tregs in the TDLNs via cDC2s. These Tregs subsequently migrate into the tumor and suppress CD8⁺ T-cell-mediated antitumor responses.^198^ RT can also induce tumor cell-derived GM-CSF to trigger monocyte accumulation within the TME and monocyte differentiation into moDCs, which, in turn, promote Treg induction.^199^ Epidermal mononuclear phagocytes, such as Langerhans cells (LCs), also contribute to Treg expansion. Upon exposure to ionizing radiation (IR), LCs upregulate cyclin-dependent kinase inhibitor 1A (CDKN1A)/p21, rapidly repairing DNA damage to resist IR-induced apoptosis, migrating to TDLNs, increasing MHC-II expression, and promoting Treg expansion.^200^ Therefore, targeting the radioresistance of LCs may increase the efficacy of RT against skin tumors. In addition to promoting Treg proliferation, RT also facilitates Treg recruitment. RT expands the population of mregDCs, which mediate Treg infiltration via CCL22. Blocking CCL22 or depleting Tregs enhances the antitumor effect of RT.^201^ However, in a murine lymphoma model, accelerated irradiation reduced the proportion of intratumoral Tregs and enhanced the antitumor activity of CD8^+^ and CD4^+^ T cells. This reduction in Treg numbers may be more critical than the increase in antitumor lymphocytes in driving enhanced antitumor responses.^202^ A postablation modulation (PAM) strategy, a strategy that combines low-dose fractionated RT with a single high-dose ablative RT, significantly reduces Treg numbers while markedly increasing the number of effector immune cells, such as CD8⁺ T cells and NK cells.^203^ Therefore, the impact of RT on the number of Tregs may vary depending on the modality of RT.

RT also affects the function of TI-Tregs. Compared with other T-cell subsets, Tregs exhibit greater resistance to radiation and may even display enhanced suppressive function.^197^ For example, RT has been found to increase the expression of CTLA-4, 4-1BB, and Helios in TI-Tregs, rendering them more suppressive than their nonirradiated counterparts.^197,204^ However, some studies have reported that RT can lead to reduced Treg function.^205,206^ These discrepancies may be attributed to differences in tumor types and radiation doses. In addition, RT elevates ROS levels within the TME, which may further affect the function of Tregs, contributing to an immunosuppressive milieu.^127,194^

Given the impact of RT on Tregs, combining RT with Treg-targeting immunotherapies may yield improved therapeutic outcomes.^207–209^ Notably, current research on the effects of RT on Tregs has been conducted primarily in murine models, with limited exploration of the underlying mechanisms involved. Moreover, variations in radiation dose and cancer type may result in distinct changes in both the abundance and function of Tregs. Therefore, more comprehensive and in-depth studies are needed to clarify these processes.

#### Tregs in chemotherapy

On the basis of their primary mechanisms of action, conventional chemotherapeutic agents can be broadly classified into six categories: (1) alkylating agents, such as cyclophosphamide, cause DNA cross-linking or breakage, disrupting replication and transcription; (2) antimetabolites, such as 5-fluorouracil, mimic nucleotides and interfere with DNA/RNA synthesis, mainly targeting cells in the S phase; (3) topoisomerase inhibitors, such as irinotecan, block DNA unwinding by inhibiting topoisomerase I or II; (4) microtubule inhibitors, such as paclitaxel and vincristine, disrupt microtubule dynamics and block mitosis; (5) antitumor antibiotics, such as bleomycin, damage DNA via intercalation or ROS generation; and (6) platinum-based drugs, such as cisplatin and carboplatin, form DNA cross-links that hinder replication.^210^ Various chemotherapy drugs can directly or indirectly affect Tregs in tumors, thereby influencing their therapeutic efficacy (Fig. 5b).

In addition to its direct cytotoxic effects, cyclophosphamide (CTX) exerts antitumor activity by directly depleting Treg numbers.^211^ Moreover, the efficacy of CTX is partially dependent on the gut microbiota. Studies have shown that *Enterococcus hirae* can translocate from the small intestine to secondary lymphoid organs, where it enhances CTX efficacy by reducing Treg abundance in the TME and increasing the CD8⁺ T-cell/Treg ratio.^212^ Low-dose metronomic CTX can deplete Treg numbers, thereby increasing the efficacy of immunotherapies, including OX40 agonists and PD-1 inhibitors.^213^ Camptothecin disrupts the stability of Foxp3 expression by inhibiting the transcriptional activity and expression of Nr4a factors. When combined with COX-2 inhibitors, it can suppress Treg function, reduce Treg abundance, and slow tumor growth.^214^ Paclitaxel has been reported to induce ferroptosis in tumor cells by increasing the intracellular levels of lipid ROS. Ferroptosis plays a positive role in modulating the TME, including reducing Treg proportions and enhancing antitumor immune responses.^215^ Gemcitabine (GEM) has also been shown to improve the immune status of patients with cancer. It significantly reduces the levels of MDSCs, Tregs, and TGF-β1 in the peripheral blood of patients with pancreatic cancer without impairing Teff function.^216^ Recent studies have systematically analyzed the Treg-specific regulatory network in tumors and identified key master regulators (MRs) responsible for Treg accumulation in the TME. Computational predictions and in vivo validation suggest that low-dose GEM is among the most promising agents for reversing MR activity, thereby depleting MR-expressing TI-Tregs, enhancing anti-PD-1 efficacy, and suppressing tumor growth.^217^ Furthermore, integrin-targeting micellar GEM and paclitaxel in combination with polymersomal CpG have been shown to significantly reduce Treg infiltration, alleviate the immunosuppressive features of the TME, and potentiate antitumor immunity.^218^ However, chemotherapeutic agents may also enhance Treg-mediated immunosuppression. For example, treatment with chemotherapeutic drugs such as cisplatin induces tumor cell apoptosis. Apoptotic tumor cells are subsequently phagocytosed by medullary sinus macrophages (MSMs) in TDLNs, leading to the production of IL-33. IL-33 subsequently activates Tregs within TDLNs and promotes their migration back to the TME, where they suppress CD8⁺ T-cell-mediated antitumor immunity, thereby impairing therapeutic efficacy and contributing to tumor resistance.^219^ Compared with chemotherapy alone, combination therapy with an anti-ST2 antibody (αST2 IgG) significantly reduces the tumor volume.^219^

Currently, studies investigating Treg alterations following chemotherapy are limited. The diversity of chemotherapeutic agents and treatment regimens further complicates this research. Therefore, more in-depth studies are needed to provide a mechanistic basis for rational combinations of chemotherapy and immunotherapy.

#### Tregs in tumor surgery

Surgical resection remains the mainstay treatment for solid tumors. However, surgery can lead to the dissemination of circulating tumor cells and trigger an inflammatory response that contributes to the formation of a supportive TME.^220^ Moreover, surgical trauma itself can induce the expansion and recruitment of various immune cells, including Tregs.^220,221^ Here, we summarize the changes in Tregs following tumor surgery and their impact on therapeutic outcomes (Fig. 5c).

Following tumor resection, Treg levels may initially decrease transiently but subsequently increase, facilitating the establishment of an immunosuppressive TME.^220^ The extent and pattern of Treg changes vary depending on the tumor type, stage, and surgical approach.^220^ Notably, compared with that of primary tumors, the TME of recurrent tumors often exhibits a pronounced expansion of the Treg population.^222^ In murine models, the expression of CCL18 is significantly upregulated in both tumor tissue and the peritoneal cavity following open abdominal surgery, promoting Treg recruitment and accelerating postoperative tumor progression.^220^ In patients with lung cancer who undergo thoracotomy, the number of monocytic MDSCs increases, which not only promotes Treg expansion but also elevates the expression of immunosuppressive molecules such as Foxp3, PD-1, and CTLA-4, thereby exacerbating immune suppression and facilitating metastasis.^223^ Additionally, surgical stress and the resulting inflammatory response can induce the release of catecholamines and prostaglandins (PGs), which increase peripheral Foxp3 levels and Treg proportions, contributing to postoperative immunosuppression. Blocking catecholamines and PGs has been shown to suppress this Treg elevation and reverse the immunosuppressive effects.^224^ Accordingly, studies have explored perioperative interventions targeting Tregs to alleviate surgery-induced immune suppression, reduce tumor recurrence and metastasis, and improve patient survival.^224–227^

### Tregs in ICB therapy

Given the critical role of immune responses in tumor progression, efforts have increasingly focused on developing immunotherapies that enhance antitumor immunity to eliminate cancer cells.^228^ Over the past decade, ICB therapy targeting T-cell checkpoint proteins, especially PD-1/PD-L1 and CTLA-4, has made remarkable progress and has been approved for the treatment of various malignancies, including melanoma, non-small cell lung cancer (NSCLC), head and neck cancer, and HCC, fundamentally reshaping treatment strategies for cancer.^228^ In addition to these classical targets, emerging checkpoints, such as LAG-3, TIM-3, and TIGIT, are under active investigation for improving therapeutic efficacy.^229,230^ However, not all patients respond favorably to immunotherapy.^228^ Subsequent studies revealed that Tregs play a pivotal role in determining the efficacy of antitumor immunotherapy (Fig. 5d‒f).^7^

#### Tregs in anti-PD-1/PD-L1 therapy

PD-1 expression increases after T-cell activation and inhibits T-cell effector functions by negatively regulating TCR signaling and costimulatory pathways. Blocking this pathway can enhance T-cell function and antigen presentation.^231,232^ Additionally, PD-1 serves as a marker of exhausted T cells (Tex cells), and inhibiting PD-1 can reactivate these “dysfunctional” CD8⁺ Tex cells.^233^ However, Tregs in the TME highly express PD-1 and are therefore also affected by anti-PD-1/PD-L1 therapy (Fig. 5d).^234^ In patients with gastric cancer, melanoma, breast cancer, or NSCLC who do not respond to anti-PD-1/PD-L1 therapy, Tregs were found to expand and enhance their immunosuppressive functions, hindering the efficacy of ICB therapy.^234–236^ The depletion of Tregs resensitizes tumors to anti-PD-1/PD-L1 therapy.^237^ Furthermore, the proportion of Tregs in patients with tumors can be used to predict the effectiveness of anti-PD-1/PD-L1 therapy.^238^ For example, an increased ratio of PD-1⁺ CD8⁺ T cells to PD-1⁺ Tregs is more likely to respond positively to anti-PD-1 therapy.^238^ In surgical tumor samples from NSCLC patients treated with anti-PD-1 therapy, researchers reported that the abundance of CCR8⁺ Tregs was associated with poor response rates.^239^ Combining Treg-targeted strategies with anti-PD-1/PD-L1 therapy may improve treatment efficacy, increase patient benefit, and enable more precise management of immunotherapy.

Several mechanisms explain how anti-PD-1/PD-L1 therapy may expand and enhance Treg function. Notably, the PD-L1-PD-1 pathway can inhibit TCR and CD28 signaling through SHP2, thereby suppressing Treg function. After anti-PD-1 treatment, TCR and CD28 signaling in PD-1⁺ Tregs within the TME is activated, enhancing downstream PI (3)K–Akt signaling and significantly increasing their immunosuppressive activity.^238^ Additionally, the number of CD8^+^ T cells increases after anti-PD-1 therapy, leading to increased IL-2 production from CD8^+^ T cells, which indirectly leads to ICOS expression in tumor Tregs and promotes their accumulation.^235^ Accordingly, researchers have reported that inhibiting ICOS signaling prior to anti-PD-1 immunotherapy significantly enhances its antitumor effects, suggesting a new strategy for cancer immunotherapy.^235^ Similarly, in HCC, researchers have reported that anti-PD-1 treatment increases IL-2 signaling, leading to the expansion of CD29^+^ Tregs with stronger immunosuppressive functions within the TME.^240^ PD-1 blockade also enhances STAT5 signaling in Tregs, leading to the upregulation of CD30 expression and promoting the suppressive function of Tregs.^241^

However, some studies have reported that PD-1 expression on Tregs helps maintain their homeostasis and function. Using anti-PD-1 antibodies or inducing PD-1 knockout weakens the stability, suppressive ability, and metabolic adaptation of TI-Tregs, thus enhancing antitumor immunity.^162,242^ Mechanistically, PD-1 downregulates the expression of the endoplasmic reticulum–lysosomal protease asparagine endopeptidase (AEP), stabilizing Foxp3 expression in Tregs.^243^ PD-1 also promotes Treg stability by enhancing fatty acid metabolism and OXPHOS while inhibiting IFN-γ and PI3K signaling.^161,162^ Notably, although PD-1⁺ Tregs are associated with a fragile phenotype, PD-1 blockade can inhibit the transendothelial migration of these PD-1⁺ fragile Tregs, leading to their retention within the tumor and contributing to tumor regression.^244^ These conflicting results may be related to the developmental stage of Tregs, the tissue microenvironment, and the timing of initial interventions. A detailed evaluation of these factors can enhance treatment efficacy.

#### Tregs in anti-CTLA-4 therapy

The anti-CTLA-4 antibody ipilimumab was the first immune checkpoint inhibitor (ICI) approved for clinical use, paving the way for the development and application of many subsequent ICIs.^245^ CTLA-4 is expressed mainly on Tregs and activated T cells. It is a homolog of CD28 but has a relatively high affinity for CD80 and CD86, thereby outcompeting CD28 for binding and delivering inhibitory signals that negatively regulate T-cell activation.^34,36,245^ While anti-CTLA-4 therapy was initially thought to restore T-cell function, recent studies have revealed that it disrupts Treg-mediated CD80/CD86 competition, impairing their immunosuppressive function.^245^ Specifically, the Fc domain of anti-CTLA-4 antibodies can interact with Fcγ receptors (FcγRs) expressed on myeloid cells or NK cells, mediating antibody-dependent cellular cytotoxicity (ADCC) to directly deplete Tregs. This mechanism increases the CD8⁺ T-cell-to-Treg ratio within the TME, thereby enhancing antitumor immunity.^246,247^ In contrast, antibodies lacking ADCC capability exhibit poor antitumor efficacy.^246^ Therefore, the therapeutic success of anti-CTLA-4 antibodies partly relies on their ability to target and eliminate Tregs (Fig. 5e).

However, although studies in mouse models have shown that anti-CTLA-4 treatment can deplete intratumoral Tregs and that therapeutic efficacy is closely associated with the extent of Treg depletion, clinical evidence suggests otherwise. Neither ipilimumab nor tremelimumab significantly reduces or depletes Foxp3⁺ cells within the TME;^248^ in some cases, Treg numbers may even increase.^249^ Research has indicated that CTLA-4 blockade can stabilize Treg‒cDC interactions, allowing Tregs to receive enhanced CD28 costimulatory signals and undergo abnormal local expansion, surpassing Teffs and counteracting the therapeutic benefits of anti-CTLA-4.^250^ To overcome this limitation, Fc-enhanced anti-CTLA-4 antibodies, such as botensilimab and XTX101, have been developed to improve ADCC–mediated depletion of Tregs.^251,252^ By increasing FcγR binding affinity, these antibodies promote deeper intratumoral Treg depletion and demonstrate superior antitumor efficacy compared with traditional anti-CTLA-4 agents.^251,252^ Moreover, CTLA-4 blockade enhances CD28 costimulation and promotes glycolysis in T cells. In a glucose-rich TME, this can destabilize Tregs and amplify antitumor immunity. Thus, combining anti-CTLA-4 antibodies with glycolysis inhibitors may represent a promising therapeutic strategy.^68^ Notably, the administration of CTLA-4-Ig following anti-CTLA-4 therapy has been shown to enhance antitumor responses. This is likely because, after initial activation, CD8⁺ T cells become less dependent on CD28 signaling for survival and function, whereas Tregs continue to rely on CD28 signaling for their maintenance and suppressive activity.^253^ Therefore, blocking CD28 signaling with CTLA-4-Ig selectively impairs Tregs without affecting CD8⁺ T cells, further enhancing antitumor immunity.^253^

#### Tregs in other types of ICB therapy

In 2022, the FDA approved the anti-LAG-3/PD-1 combination drug Opdualag for the treatment of unresectable or metastatic melanoma, thus making LAG-3 the third immune checkpoint clinically recognized after CTLA-4 and PD-1.^229,230^ Under physiological conditions, LAG-3 is expressed on activated lymphocytes, B cells, NK cells, and DCs.^254^ LAG-3 exerts its effects through interactions with ligands such as MHC-II and FGL-1.^254^ In conventional T cells (Tconvs), the binding of LAG-3 to these ligands suppresses their proliferation and effector functions.^255^ Notably, LAG-3 has a much greater affinity for MHC-II than does CD4, enabling it to modulate CD4⁺ T-cell responses by competitively binding MHC-II.^254^ Unlike Tconvs, Tregs express LAG-3 to increase their stability and suppressive function.^146^ Therefore, anti-LAG-3 therapy may also influence both the number and function of Tregs. Studies have shown that combined anti-LAG-3/PD-1 treatment is more effective than anti-PD-1 therapy alone.^229,256^ Additionally, LAG-3 blockade disrupts the potent immunosuppressive activity of Tregs coexpressing OX40, glucocorticoid-induced tumor necrosis factor receptor (GITR), and LAG-3.^185^ Moreover, in blood samples from patients with melanoma, combined anti-LAG-3/PD-1 treatment has been shown to reduce the metabolic activity of Tregs and impair their immunosuppressive function.^257^ These findings suggest that the antitumor efficacy of LAG-3 blockade is partly mediated through its impact on Tregs.

TIM-3 is an immune checkpoint protein with inhibitory functions that was originally identified on Th1 cells, CD8⁺ T cells, and Tregs, particularly as a hallmark of terminally Tex cells.^229,230^ TIM-3 is also expressed by myeloid cells, including macrophages, DCs, and NK cells, as well as some tumor cells.^230,258^ Its known ligands include galectin-9 (Gal-9), high-mobility group box 1 (HMGB1), phosphatidylserine (PtdSer), and carcinoembryonic antigen-related cell adhesion molecule 1 (CEACAM-1).^230^ TIM-3 was initially identified as an inhibitory receptor on Teffs but is now recognized as a key regulator of both Treg and myeloid cell functions.^258^ Specifically, it promotes the effector phenotype and suppressive function of Tregs while inhibiting inflammasome (NLRP3) activation in DCs.^259,260^ In mouse models, targeting TIM-3 reduces Treg abundance and alleviates Treg-mediated immunosuppression.^261^ Notably, TIM-3⁺Foxp3⁺ Tregs (TFTs) are enriched in certain tumor types and are associated with poor prognosis, suggesting that anti-TIM-3 antibodies could serve as promising therapeutic agents in such contexts.^259,262,263^

TIGIT, identified in 2009, is a novel coinhibitory molecule expressed on Tregs, memory T cells, and natural killer (NK) cells. It is also transiently upregulated in T cells upon TCR stimulation.^230^ The main ligands of TIGIT are CD155 and CD112, which are broadly expressed on APCs as well as on various nonhematopoietic cell types, including tumor cells. Additional ligands include CD96, CD113, nectin-4, and Fab2.^229,230^ By engaging with their ligands, TIGIT modulates immune cell function and immune responses, generally promoting an immunosuppressive state, thus making it a potential target for antitumor immunotherapy.^264,265^ In Tregs, TIGIT is a direct target of Foxp3 and serves as a marker of suppressive function.^266^ TIGIT can induce the expression of the immunosuppressive factor Fgl2 and selectively inhibit Th1 and Th17 responses.^267^ Notably, TIGIT expression is significantly elevated within TI-Tregs, highlighting its functional importance within the TME.^268^ In mouse models of NSCLC, HNSCC, and ovarian cancer, anti-TIGIT therapy downregulates the expression of Treg-associated genes, weakens Treg-mediated immunosuppression, and improves tumor outcomes.^269–271^ Clinical studies have shown that in NSCLC, prognosis following combination therapy with the TIGIT antibody tiragolumab and the PD-L1 antibody atezolizumab positively correlates with baseline levels of intratumoral macrophages and Tregs (Fig. 5f).^269^

### Targeting Tregs for tumor therapy

Given their pivotal role in tumor progression, Tregs have become a key target in cancer immunotherapy.^7,13^ Treg-targeted strategies show great potential when combined with various therapeutic approaches. Current strategies mainly aim to reduce their intratumoral abundance, target Treg-associated effector molecules, or impair their suppressive function. In particular, focusing on molecules preferentially expressed by TI-Tregs may help minimize systemic immune disruption and reduce adverse effects. Table 2 provides a summary of ongoing and completed clinical trials targeting Tregs for cancer therapy for reference.

**Table 2:** Clinical trials targeting Tregs in cancer therapy

Target	Cancer type	Drug	Combination therapy	Phase	Status	NCT number
CD25	Melanoma	Daclizumab	Dendritic cell-based vaccines	1/2	Completed	NCT00847106
	Metastatic melanoma	RFT5pdgA	–	2	Completed	NCT00314093
	Solid tumors	E7777	Pembrolizumab	1/2	Recruiting	NCT05200559
	DLBCL	E7778	–	1/2	Recruiting	NCT04855253
CCR4	Solid tumors	Mogamulizumab	Nivolumab	1	Completed	NCT02946671
	ATL, PTCL	KW-0761	–	1	Completed	NCT00355472
CCR8	Solid tumors	CHS-114	Toripalimab	1	Recruiting	NCT05635643
	Solid tumors	BAY3375968	Pembrolizumab	1	Recruiting	NCT05537740
	TNBC	LM-108	Toripalimab, Eribulin, Nab-paclitaxel	2	Recruiting	NCT06387628
	Solid tumors	CHS-114	Toripalimab, 5-FU, Cisplatin	1	Recruiting	NCT06657144
	Solid tumors	GS-1811	Zimberelimab	1	Recruiting	NCT05007782
	Solid tumors	S-531011	Pembrolizumab	1/2	Recruiting	NCT05101070
	Solid tumors	LM-108	Toripalimab	2	Recruiting	NCT06873854
	Solid tumors	AMG 355	Pembrolizumab	1	Recruiting	NCT06131398
	NSCLC	LM-108	Sintilimab	2	Not yet recruiting	NCT06620822
	Solid tumors	LM-108	Toripalimab	1/2	Enrolling by Invitation	NCT05518045
TGF-β	MPM	GC1008	–	2	Completed	NCT01112293
	Solid tumors	TEW-7197	–	1	Completed	NCT02160106
	Desmoid tumor	Vactosertib	Imatinib	1/2	Completed	NCT03802084
	Advanced tumors	AK130	AK130 simultaneously targets TIGIT	1	Completed	NCT05653284
	Pancreatic cancer	HCW9218	IL-15 Protein	1/2	Active, not recruiting	NCT05304936
	Solid tumors	HCW9219	IL-15 Protein	1	Active, not recruiting	NCT05322408
	Breast cancer	Losartan	–	2	Recruiting	NCT05637216
	Solid tumors	HB0028	HB0028 simultaneously targets PD-L1	1/2	Recruiting	NCT06223308
	Solid tumors	ES014	ES014 simultaneously targets CD39	1	Recruiting	NCT05717348
	Pancreatic cancer	Losartan	Folfirinox, 9-ING-41	2	Recruiting	NCT05077800
	HNSCC	Ficerafusp alfa	Pembrolizumab; ficerafusp targets EGFR	2/3	Recruiting	NCT06788990
IL-10	Solid tumors	LB4330	LB1410	1/2	Recruiting	NCT06468358
CD39	Solid tumors	TTX-030	Pembrolizumab, Gemcitabine, Nab-paclitaxel	1	Completed	NCT03884556
	Solid tumors	TTX-030	Budigalimab, mFOLFOX6, Docetaxel, etc.	1	Completed	NCT04306900
	PDAC	SRF617	Gemcitabine, Albumin-bound Paclitaxel, Pembrolizumab	1	Completed	NCT04336098
	Solid tumors	ES002023	–	1	Completed	NCT05075564
CD73	Solid tumors	AK119	AK104	1	Completed	NCT04572152
	Solid tumors	AK119	–	1	Completed	NCT05173792
	PDAC	Oleclumab	Durvalumab + chemotherapy	–	Completed	NCT03611556
	Solid tumors	Sym024	Sym021	1	Completed	NCT04672434
	Advanced tumors	CPI-006	Ciforadenant, Pembrolizumab	1	Completed	NCT03454451
	Solid tumors	LY3475070	Pembrolizumab	1	Completed	NCT04148937
	Solid tumors	JAB-BX102	Pembrolizumab	1/2	Recruiting	NCT05174585
	Solid tumors	PM1015	–	1	Recruiting	NCT05950815
	Solid tumors	AK131	AK131 simultaneously targets PD-1	1	Recruiting	NCT06166888
	Solid tumors	IPH5301	Chemotherapy and Trastuzumab	1	Recruiting	NCT05143970
	Solid tumors	BR101	–	1	Recruiting	NCT06001580
	Advanced tumors	AK137	AK137 simultaneously targets LAG-3	1	Not yet recruiting	NCT06691360
A2AR	Multiple myeloma	Ciforadenant	Daratumumab	1	Completed	NCT04280328
	NSCLC	PBF-509	PDR001	1	Completed	NCT02403193
	Prostate cancer	AZD4635	Oleclumab, Durvalumab	2	Completed	NCT04089553
	Advanced tumors	Ciforadenant	Atezolizumab	1	Completed	NCT02655822
	mCRPC	Etrumadenant	Zimberelimab, Enzalutamide, etc.	1/2	Completed	NCT04381832
	Solid tumors, NHL	AZD4635	Oleclumab, Osimertinib	1/2	Active, not recruiting	NCT03381274
	RCC	Ciforadenant	Nivolumab, Ipilimumab	1/2	Recruiting	NCT05501054
	Solid tumors	ILB-2109	Toripalimab	1/2	Recruiting	NCT05955105
Nrp1	Solid tumors	MNRP1685A	–	1	Completed	NCT00747734
HDAC	Breast cancer	Entinostat	Ipilimumab, Nivolumab	1	Active, not recruiting	NCT02453620

*DLBCL* diffuse large B-cell lymphoma, *ATL* adult T-cell leukemia, *PTCL* peripheral T-cell lymphoma, *TNBC* Triple-Negative Breast Cancer, *NSCLC* non-small cell lung cancer, *MPM* Malignant pleural mesothelioma, *HNSCC* head and neck squamous cell carcinoma, *PDAC* pancreatic ductal adenocarcinoma, *mCRPC* Metastatic castration-resistant prostate cancer, *EGFR* epidermal growth factor receptor, *HDAC* histone deacetylase

#### Promotion of Treg depletion

CD25, the α-chain of the heterotrimeric IL-2 receptor, is expressed primarily on Tregs and activated T cells and is considered a hallmark of Tregs, making it a potential target for Treg depletion.^272^ Therapeutic strategies targeting CD25 have focused mainly on blocking IL-2 signaling or eliminating CD25-expressing cells. However, early studies using anti-CD25 antibodies or IL-2–diphtheria toxin fusion proteins for cancer treatment yielded suboptimal results, as these approaches either failed to effectively deplete intratumoral Tregs or concomitantly eliminated antitumor T cells.^273,274^ Subsequent research optimized FcγR binding to enhance ADCC, which significantly improved intratumoral Treg depletion, increased the Teff/Treg ratio, and improved tumor prognosis.^275^ Notably, combining this approach with anti-PD-1 antibodies has demonstrated synergistic therapeutic effects.^275^ In addition, non-IL-2-blocking anti-CD25 antibodies have been developed to selectively deplete Tregs while preserving Teffs, resulting in superior antitumor efficacy.^276^ Future efforts should refine anti-CD25 therapies to minimize Teff impacts and optimize dosing and timing.

GITR is constitutively expressed on Tregs. Upon activation, it can induce Treg instability and functional suppression and even promote their conversion into cytotoxic Th1-like cells.^277,278^ In contrast, GITR is also expressed on activated CD4⁺ Tconvs and CTLs, where its activation enhances their proliferation and effector functions.^279^ In various murine tumor models, agonistic anti-GITR antibodies suppressed Treg function and reduced intratumoral Treg numbers through FcγR-mediated ADCC.^278,280,281^ Future research should focus on developing novel antibodies and exploring combination strategies with other immunotherapies.^278,282,283^ For example, a fusion protein comprising an anti-PD-1 antibody and GITR ligand (GITR-L) can promote GITR clustering, thereby enhancing and sustaining GITR-L-mediated T-cell activation, proliferation, and differentiation of antigen-specific memory T cells while also reducing the number of TI-Tregs and alleviating Treg-mediated immunosuppression.^283^

4-1BB (also known as CD137/TNFRSF9) is a membrane glycoprotein, and TI-Tregs in various cancers commonly express 4-1BB.^284,285^ Targeting 4-1BB can effectively deplete Tregs expressing this receptor.^286^ In addition, 4-1BB activation promotes the proliferation, survival, and effector functions of CD4⁺ and CD8⁺ T cells and induces the production of IFN-γ.^286–288^ However, potent agonistic anti-4-1BB antibodies have shown suboptimal results in clinical studies, and their hepatotoxicity further limits their use. Therefore, novel antibodies are being developed to reduce toxicity while improving efficacy.^289^ Recent studies have developed weak agonistic 4-1BB antibodies fused with IL-15 precursors, which selectively deplete Tregs and expand activated CD8^+^ T cells at tumor sites. This approach achieves targeted release of IL-15 within the TME, reduces toxicity, and opens new avenues for the development of 4-1BB-targeted therapy.^290^

#### Reducing Treg recruitment

Tregs are recruited to the TME through various chemokine–chemokine receptor interactions. Among them, the CCL17/CCL22–CCR4 and CCL1–CCR8 axes stand out as the most promising therapeutic targets.^7,111^ CCL17 and CCL22 recruit Tregs into the TME via a CCR4-dependent mechanism, promoting tumor immune evasion.^291,292^ Furthermore, CCR4 is expressed primarily on effector Tregs, and CCR4⁺ Tregs are more immunosuppressive than CCR4⁻ Tregs.^293^ Therefore, targeting CCR4 is more effective in reducing the number of Tregs that play a critical role in the tumor immune microenvironment.^293^ Chemokine receptor 8 (CCR8) is also expressed predominantly on effector Tregs in tumors, with limited expression on peripheral Tregs, making it a specific target for tumor Tregs.^111^ CCL1, the main ligand for CCR8, not only induces Treg migration but also promotes Treg differentiation and the expression of effector molecules such as CD39, IL-10, and granzymes.^112,294^ In bladder cancer and HCC models, CCR8 blockade has been shown to convert tumor-infiltrating Tregs into an unstable, fragile phenotype with reduced suppressive ability.^295,296^ Furthermore, targeting CCR4 and CCR8 not only reduces the recruitment of Tregs but also enables the direct elimination of effector Tregs expressing these chemokine receptors through the ADCC, representing highly promising therapeutic strategies for TI-Tregs.^297,298^ Currently, several monoclonal antibodies or antagonists targeting CCR8 or CCR4 are undergoing clinical trials (Table 2), and their safety and efficacy as monotherapies or in combination therapies are being assessed, with promising preliminary results.^297–300^ Future research should explore their expression mechanisms, functions, and therapeutic potential across diverse tumor types.

#### Targeting Treg effector molecules

Tregs exert their immunosuppressive functions by secreting a variety of cytokines, including TGF-β, IL-10, and IL-35.^38–40^ Among these cytokines, TGF-β not only plays an immunosuppressive role but is also essential for Treg differentiation.^20^ In animal tumor models, TGF-β inhibitors have shown promising antitumor effects both as monotherapies and in combination with immunotherapy.^301,302^ However, clinical trials targeting TGF-β have yielded limited success and, in some cases, even promoted tumor progression and induced cardiovascular adverse events.^303^ These discrepancies may be attributed to the complex and context-dependent roles of TGF-β in both physiological and cancer settings.^303^ Future research should elucidate TGF-β signaling mechanisms and develop precise modulation strategies. As mentioned previously, Tregs specifically express GARP and integrin αvβ8 to activate TGF-β, making them potential therapeutic targets.^124,125^ IL-10 is another possible target; however, IL-10 plays a paradoxical role in the TME.^304^ While it may suppress antigen presentation by APCs and inhibit the production of proinflammatory cytokines, it can also promote the proliferation and cytotoxic activity of CTLs.^304^ These dual roles complicate its therapeutic targeting, and the outcome may depend heavily on the specific tumor context. Furthermore, IL-10 blockade can significantly impact peripheral immune tolerance, limiting its clinical application.^305,306^ IL-35, a primarily Treg-derived immunosuppressive cytokine, is known to promote tumor growth and metastasis.^39^ Although some studies have demonstrated that limiting IL-35 within tumors can effectively suppress tumor progression,^307^ IL-35-targeted therapies are still in the early stages, and further exploration of IL-35-related signaling pathways and the development of monoclonal antibodies are needed.

Tregs express CD39 and CD73, which convert extracellular ATP/ADP into adenosine, thereby exerting immunosuppressive effects through binding to its receptor A2AR.^37,127^ As such, CD39, CD73, and A2AR are promising therapeutic targets and have demonstrated favorable outcomes in preclinical studies. Currently, multiple monoclonal antibodies and small-molecule inhibitors targeting this pathway have entered clinical trials to evaluate their efficacy and safety as monotherapies or in combination with ICB therapy (Table 2). For example, the A2AR antagonist AZD4635 is being tested as a monotherapy or in combination with the PD-L1 inhibitor durvalumab in patients with metastatic castration-resistant prostate cancer. The combination therapy induced partial responses in some cases and showed good tolerability, supporting further phase II trials in this population.^308^ Overall, current research on targeting CD39, CD73, and A2AR in cancer therapy remains promising and may lead to novel strategies for cancer immunotherapy.

#### Modulating Treg stability

Given the critical role of Tregs in maintaining immune homeostasis and suppressing autoimmune diseases, their depletion may lead to severe side effects. Therefore, modifying the stability of Tregs in tumors, promoting their conversion into unstable or fragile Tregs, is an ideal therapeutic approach. As discussed earlier, many mechanisms by which Tregs maintain stability in tumors have been identified, and researchers have discovered several potential therapeutic targets on the basis of these mechanisms. For example, targeting the Nrp-1-SEMA4A axis can promote the transition of Tregs to fragile Tregs;^74,309^ altering the DNA hypomethylation status and histone acetylation status of *Foxp3* can impact its transcription;^310,311^ targeting metabolism-related molecules such as MCT-1 and CD36 can shift the metabolic state of Tregs in tumors, promoting an unstable phenotype;^67,119,164^ and inhibiting deubiquitinases such as USP7 and USP47 and activating the E3 ligase STUB1 can suppress *Foxp3* transcription or promote Foxp3 ubiquitin-mediated degradation, weakening Treg stability.^64,312,313^ Although research on targeting Treg stability is still largely in its basic stages, with insufficient clinical studies to validate its efficacy and safety, this field shows great potential for future applications.

## Tregs in inflammation

Inflammation is an adaptive response triggered by harmful stimuli and pathological conditions such as infection and tissue injury.^314^ A successful inflammatory response typically results in the clearance of pathogens and necrotic tissue, followed by the resolution of inflammation and the initiation of tissue repair.^315^ However, when harmful stimuli persist, or the inflammatory response fails to resolve properly, it may progress to chronic inflammation, as observed in autoimmune reactions driven by sustained exposure to self-antigens.^314,316^ In addition, certain systemic chronic inflammatory states, such as atherosclerosis, obesity, and asthma, are not initiated by classical inflammatory triggers such as infection or injury but are instead closely associated with disruptions in tissue homeostasis.^314^ Tregs serve as key negative regulators of inflammation. They not only suppress the activation of autoreactive T cells but also selectively permit beneficial immune responses.^317^ Conversely, quantitative or functional abnormalities in Tregs can lead to unresolved inflammation and tissue damage, thereby promoting the initiation and progression of chronic inflammatory diseases.^9^

### Tregs in autoimmune disease

**Fig. 6:**
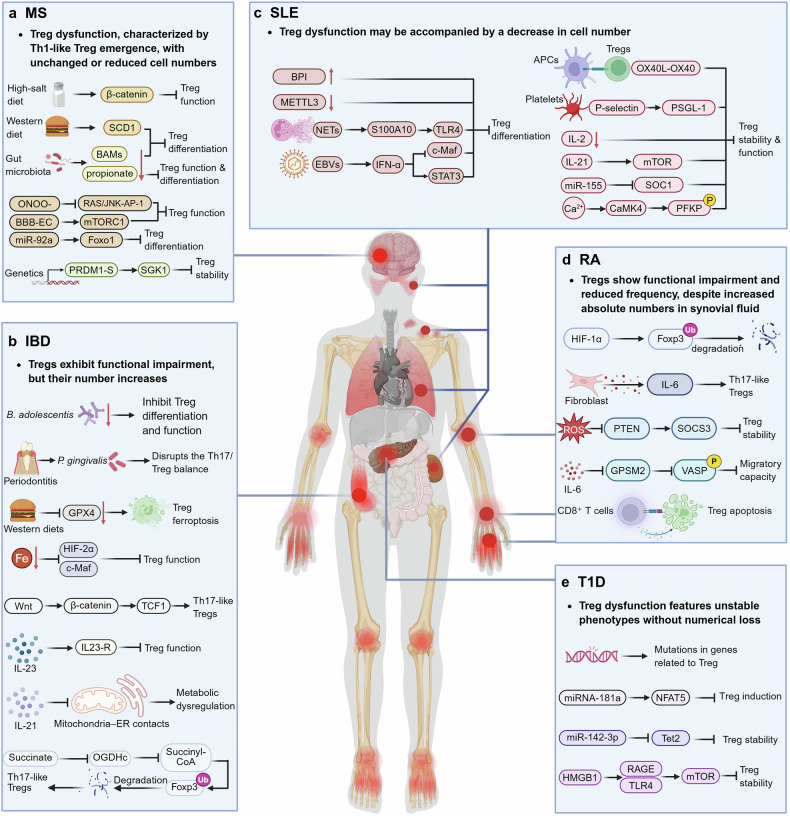
Dysregulation of Tregs in autoimmune diseases. In autoimmune diseases, the number, proportion, and function of Tregs are altered. **a** In MS, unhealthy dietary habits, altered gut microbiota, inflammatory microenvironments, and genetic/epigenetic changes influence Treg differentiation, stability, and function. **b** In IBD, Treg imbalance and dysfunction are driven mainly by the gut microbiota and dietary factors; additionally, intestinal environmental signals such as Wnt, IL-23, and IL-21 may influence Treg stability and suppressive capacity. **c** In SLE, Treg differentiation defects are associated with dysregulated expression of BPI, METTL3, NETs, and Epstein–Barr virus infection. Their function is further impaired by inflammatory cues, including reduced IL-2 and elevated IL-21. Increased intracellular calcium levels may also impair Treg stability and function. **d** In RA, the hypoxic synovial microenvironment, excessive ROS, excessive IL-6, and direct cytotoxicity from CD8⁺ T cells negatively affect Tregs. **e** In T1D, Treg dysfunction is attributed to genetic mutations, while certain microRNAs and HMGB1 released from pancreatic β cells also contribute. This figure was created with Biorender.com

Autoimmune diseases, such as multiple sclerosis (MS), inflammatory bowel disease (IBD), systemic lupus erythematosus (SLE), rheumatoid arthritis (RA), and type 1 diabetes (T1D), are characterized by aberrant immune responses targeting host cells, tissues, and organs, leading to tissue damage and inflammation. An imbalance between Tregs and proinflammatory cells, along with the impaired immunosuppressive function of Tregs, contributes substantially to the pathogenesis and progression of these disorders, as Tregs fail to effectively restrain autoreactive immune responses.^8,9^ In recent years, advancements in emerging technologies have facilitated a broader understanding of the heterogeneity of Tregs and the molecular mechanisms underlying their dysregulation in autoimmune diseases (Fig. 6).

#### Tregs in MS

MS is a chronic disease that affects the central nervous system, particularly the brain and spinal cord. It is characterized by the attack of T lymphocytes specific to neuronal antigens on the myelin sheath—the protective lipid layer around nerve cells—causing damage to the myelin structure, resulting in impaired nerve signal transmission and various symptoms, such as visual disturbances, limb weakness or numbness, balance and coordination difficulties, fatigue, and cognitive impairment.^318^ Th1 and Th17 cells are considered major pathogenic cells that cause chronic inflammation in the nervous system by generating various cytokines, including IFN-γ, IL-17, and TNF-α.^319^ In patients with MS, the number of Tregs is either decreased or unchanged, but functional defects in these cells are commonly reported.^186,320–322^ Notably, multiple studies have reported an increase in IFN-γ–expressing Th1-like Tregs, which exhibit a fragile Treg phenotype, in patients with MS. This leads to the overactivation of Th1 and Th17 cells and results in excessive secretion of inflammatory cytokines.^321,323^ The causes of Treg alterations in MS remain unclear, but they may be associated with factors such as diet, gut microbiota dysbiosis, environmental triggers, and genetic predispositions (Fig. 6a).

A high-salt diet has been associated with an increased risk of MS. A high salt concentration promotes the differentiation of naïve T cells into Th17 cells and compromises Treg stability, as indicated by aberrant IFN-γ expression.^324,325^ Moreover, a high-salt diet induces abnormal activation of β-catenin in Tregs, which, through a positive feedback loop involving the prostaglandin E2 receptor (PTGER)–β-catenin axis and the β-catenin–serum/glucocorticoid-regulated kinase 1 (SGK1)–Foxo pathway, results in the loss of immunosuppressive function and an imbalance between IFN-γ and IL-10.^323^ A high-fat or Western diet also promotes the expression of stearoyl-CoA desaturase-1 (SCD1), thereby inhibiting Treg differentiation and exacerbating MS.^326^

Secondary bile acid metabolites (BAMs) promote the differentiation of Foxp3⁺ Tregs and suppress Th17 cell development;^327^ however, in patients with MS, the abundance of gut bacteria responsible for generating BAMs is significantly reduced, contributing to immune imbalance.^328^ In addition, SCFA-producing bacteria such as *Butyricimonas* are decreased in the gut of patients with MS, leading to reduced propionate levels, which are accompanied by diminished Treg numbers and impaired function.^329^ Propionate supplementation has been shown to restore Treg abundance, improve Treg metabolism and function, and alleviate disease severity in MS.^329^

The disease microenvironment also plays a crucial role in modulating Treg function. Disruption of redox homeostasis in the central nervous system is implicated in the pathogenesis of MS.^318^ Peroxynitrite, formed by the reaction between nitric oxide (·NO) and superoxide anion (O₂·⁻), can induce nitration of the IL-2 receptor and downregulate the RAS/JNK–AP-1 signaling pathway, thereby impairing Treg function and disrupting the Treg/Th17 balance.^330^ Additionally, the interaction between Tregs and inflamed blood–brain barrier endothelial cells upregulates the mTORC1 signaling pathway, causing the loss of Treg function and the acquisition of Th1/17 characteristics.^331^ Treatment with rapamycin can reverse this process.^331^ Serum levels of miR-92a are significantly elevated in patients with MS. By targeting Foxo1, miR-92a promotes Th17 differentiation while suppressing Treg differentiation and immunosuppressive function, thereby driving central nervous system autoimmunity.^332^

Finally, genetic and epigenetic alterations can also lead to Treg dysfunction and contribute to the progression of MS.^333^ For example, comprehensive transcriptomic and epigenomic profiling of Tregs from patients with MS revealed the upregulation of a short splice isoform of *PRDM1*—which encodes B lymphocyte-induced maturation protein 1 (BLIMP1). This isoform induces the expression of SGK1, resulting in Foxp3 instability and subsequent Treg dysfunction.^334^

Overall, Tregs in patients with MS often exhibit functional impairment and phenotypic instability, driven by a combination of contributing factors. Epidemiological studies have identified several high-risk factors, such as high-salt diets, vitamin D deficiency, Epstein–Barr virus infection, and gut microbiota dysbiosis,^318,324,329^, which are closely associated with increased MS susceptibility. Notably, these factors may also disrupt Treg differentiation, function, or stability, thereby promoting disease progression. Future studies should focus on the spatiotemporal dynamics of Treg dysregulation. By integrating single-cell technologies, multiomics approaches, and human tissue samples, researchers can systematically uncover the key mechanisms underlying Treg instability and identify more precise, personalized therapeutic targets.

#### Tregs in IBD

IBD is a gut disorder caused by abnormal immune system responses and presents as Crohn’s disease (CD) and ulcerative colitis (UC). A notable feature of IBD is the accumulation of proinflammatory T cells and cytokines in the gut tissue due to impaired mucosal barrier function, along with abnormal immune responses to the gut microbiota.^335,336^ The pathogenesis of IBD is highly complex and involves a multifactorial interplay between genetics, the gut microbiota, the diet, and the immune system.^336^ Although the Treg population in the inflamed tissues of patients with IBD increases, possibly through feedback regulation owing to excessive inflammation, the ratio between Tregs and Th17 cells decreases.^337^ Tregs exhibit marked functional heterogeneity in IBD. Notably, they play a critical role in maintaining intestinal homeostasis. For example, Treg-derived IL-10 can effectively suppress the onset and progression of colitis.^305,306,338^ The surface molecule LAG-3 on Tregs binds to MHC-II molecules to inhibit the activity of CX3CR1⁺ macrophages, thereby reducing the production of IL-23 and IL-1β and preserving the mucosal immune balance.^35^ In addition, intestinal Tregs specifically secrete IL-27, which limits Th17 cell-mediated inflammatory responses.^339^ However, studies have also revealed that a subset of Tregs exhibit functional impairment or dysregulation in the IBD setting (Fig. 6b). For example, a novel proinflammatory Foxp3⁺ T-cell subset highly sensitive to TNF-α signaling was identified in patients with CD.^340^ Additionally, multiple studies have identified Treg subsets exhibiting Th1-like and Th17-like features in IBD, suggesting functional abnormalities and even potential pathogenicity.^341–343^ Other studies have proposed that, within the intestinal microenvironment, TGF-β induces the expression of Areg in Tregs via Smad3 signaling, thereby exacerbating colonic fibrosis.^344^

Various risk factors for IBD influence Treg differentiation and function, including alterations in the gut microbiota. For example, the level of *Bifidobacterium adolescentis* in the feces of patients with IBD is significantly lower than that in healthy individuals, and supplementation with *B. adolescentis* can increase the proportion of Tregs and the levels of anti-inflammatory cytokines such as IL-10, IL-4, and IL-5, thereby alleviating disease severity.^345^ Moreover, periodontitis has been reported to be closely associated with IBD. The periodontal pathogen *Porphyromonas gingivalis* inhibits linoleic acid production, disrupts the Th17/Treg balance, and aggravates colitis.^346^ In addition to compositional changes in the gut microbiota, phase variation in specific bacterial genes may also influence Treg abundance. For example, in patients with IBD, *Bacteroides fragilis* exhibits phase variation that downregulates the synthesis of capsular polysaccharide A, which has been linked to reduced colonic Treg populations in the host.^347^

In addition to the gut microbiota, diet influences Tregs in patients with IBD. High fat intake can exacerbate intestinal inflammation by inducing ferroptosis in intestinal Tregs through downregulation of GPX4 expression.^348^ Similarly, the consumption of high levels of high-fructose corn syrup alters gut microbial communities, affecting the Th17 cell/Treg balance and promoting intestinal inflammation.^349^ Iron deficiency is the most prevalent extraintestinal symptom in patients with IBD. Decreased intracellular iron levels in Tregs result in the downregulation of HIF-2α and c-Maf, impairing Treg function.^350^

The inflammatory milieu further induces Treg dysfunction. In patients with IBD, inflamed mucosal tissue secretes abundant active Wnt, resulting in aberrant β-catenin activation, which promotes T-cell activation and the expression of Th17-associated effector molecules in Tregs through TCF1.^351^ In contrast to the stabilizing effect of the IL-23/IL-23R axis on the Treg phenotype in tumors, IL-23 exerts a negative regulatory effect on Tregs in the gut, promoting chronic inflammation in patients with IBD. This finding highlights that the same molecule can exert remarkably distinct effects on Tregs depending on the microenvironment.^352^ IL-21 can impair mitochondria–ER contacts in Tregs from patients with CD, driving them into a hypermetabolic state and enhancing their proinflammatory responses.^353^ The level of succinate, an immunoregulatory metabolite, is elevated in the intestines of patients with IBD. Elevated succinate downregulates the expression of genes encoding components of the OGDH complex, thereby suppressing the synthesis of succinyl-CoA. The reduction in succinyl-CoA decreases the succinylation of specific FOXP3 lysine residues, thereby promoting their ubiquitination-mediated degradation and leading Treg cells to acquire a Th17-like phenotype with impaired immunosuppressive function.^354^ Furthermore, the lamina propria serves as a key niche supporting effector Treg function; thus, its disruption may impair Treg activity.^355^

Tregs play a complex and essential regulatory role in IBD. Although their numbers are often increased in inflamed tissues, their function is frequently impaired, and they may acquire proinflammatory phenotypes. Moreover, the gut serves as a critical site for the induction and development of pTregs, and alterations in its local environment may profoundly affect systemic immune homeostasis through immunological or metabolic axes.^356–358^ Therefore, more systematic mechanistic studies are warranted to uncover potential therapeutic targets.

#### Tregs in SLE

SLE is a multisystem autoimmune disease. It is characterized by the polyclonal activation of B and T cells, the production of autoantibodies against nuclear antigens, and the formation of immune complexes. These events trigger immune-mediated inflammation, ultimately leading to severe damage to organs such as the skin, joints, lungs, and kidneys.^359^ Although the exact pathogenesis remains unclear, an imbalance between proinflammatory CD4⁺ T cells and Tregs underlies the development of SLE,^360^ studies have also reported that the number of Tregs in patients with SLE is reduced and that these cells are functionally impaired.^8,9^

First, multiple factors contribute to impaired Treg differentiation in patients with SLE. Bactericidal/permeability-increasing proteins are highly expressed in T cells and their exosomes from patients with SLE, which suppresses Treg differentiation.^361^ In addition, methyltransferase-like 3 (METTL3) is defective in CD4⁺ T cells from patients with SLE, which reduces Foxp3 expression in a m6A-dependent manner and inhibits Treg differentiation.^362^ In SLE, neutrophil-derived NETs can also suppress Treg differentiation by activating the TLR4 signaling pathway in naïve CD4⁺ T cells through S100A10.^363^ Epstein–Barr virus infection, a known risk factor for SLE, elevates IFN-α levels, which activate STAT3 and inhibit the expression of c-Maf, thereby disrupting the balance between Tregs and Th17 cells.^364^

In addition to impaired differentiation, a variety of mechanisms contribute to the decreased stability and dysfunction of Tregs in SLE. For example, APCs derived from patients with active SLE induce Treg dysfunction via the OX40L/OX40 signaling axis by downregulating Foxp3 expression.^365^ Moreover, activated P-selectin on the surface of platelets in patients with SLE binds to P-selectin glycoprotein ligand-1 modified with CD15s on Tregs, leading to the upregulation of spleen tyrosine kinase-dependent calcium signaling and the inhibition of the TGF-β pathway, thereby impairing Treg function.^366^ IL-2 plays a key role in the differentiation and phenotypic stability of Tregs. Patients with SLE have lower IL-2 levels and significantly reduced expression of CD25 in T cells, which may be a significant cause of Treg instability and impaired function.^367,368^ Conversely, IL-21 impairs the development and function of Tregs in SLE by activating the mTOR signaling pathway.^369^ Additionally, the upregulation of type I interferon signaling in Tregs from patients and mice with SLE may lead to their exhaustion and inhibit their function.^9^ Inflammation-induced microRNA-155 (miR-155) also disrupts the stability and function of Tregs by reducing the level of suppressor of cytokine signaling 1 (SOCS1).^370^ Therefore, targeting miR-155 could be a potential strategy to alleviate SLE progression.^370^ Calcium levels in T cells from patients with SLE are greater than those in healthy individuals.^368^ Tregs in SLE are characterized by increased calcium/calmodulin-dependent protein kinase 4 (CaMK4) activity, which phosphorylates downstream PFKP to promote glycolysis, resulting in metabolic and functional defects in Tregs.^371^ Finally, genetic variations in the *Foxp3* gene may also impair the immunosuppressive function of Tregs, contributing to the development and progression of SLE (Fig. 6c).^372^

SLE is an autoimmune disease that affects multiple organs and systems, yet current research has focused primarily on peripheral blood samples from patients.^359^ Owing to the difficulty in accessing certain inflamed tissues, our understanding of the abundance, phenotype, and functional status of Tregs across different tissues remains limited, and the impact of distinct inflammatory microenvironments on Tregs also remains unclear.^373^ Future studies should integrate advanced technologies such as single-cell sequencing and spatial transcriptomics to characterize the dynamic changes and functional features of Tregs at various inflamed sites systematically, thereby laying the foundation for more precise therapeutic interventions.

#### Tregs in RA

RA is a chronic, systemic autoimmune disease primarily characterized by symmetric polyarthritis, commonly affecting the small joints of the hands and feet. It can lead to severe disability and increased mortality.^374^ The pathogenesis of RA is complex, and a reduced proportion or functional impairment of Tregs, resulting in the breakdown of self-tolerance, is considered one of the potential mechanisms driving disease progression.^375^ An integrative analysis of expression quantitative trait loci (eQTLs) in immune cells and genome-wide association studies (GWASs) revealed a link between effector Tregs and RA, suggesting that Tregs are involved in the pathogenesis of RA.^376^ Studies investigating Tregs in the peripheral blood and synovial fluid of patients with RA have yielded conflicting results regarding Treg numbers and phenotypic characteristics (Table 3).^375^ The discrepancies among these findings may stem from inconsistencies in Treg identification criteria, the limited resolution of conventional analytical methods, and the overlooked intrinsic heterogeneity within Tregs. Single-cell technologies offer promising solutions to these challenges. For example, an integrated analysis of high-dimensional mass cytometry and single-cell RNA sequencing data from a clinical trial of conventional synthetic disease-modifying antirheumatic drug (csDMARD) withdrawal revealed that, during disease relapse in patients with RA, the number of circulating memory Tregs increased, but their suppressive function was impaired.^377^ Overall, many studies suggest that Tregs in RA exhibit impaired suppressive function and are often accompanied by a dysregulated Th17/Treg ratio.

**Table 3:** Summary of Treg abundance and functional status in RA

Sampling site	Treg identification markers	Treg number/proportion	Treg functional status	Ref (PMID)
Peripheral blood	CD4⁺CD25⁺	—	Impaired function	15280421
Peripheral blood	CD4⁺CD25^high^	Unchanged proportion	—	16539816
Peripheral blood	CD4⁺CD25^high^	Increased number and proportion	Impaired function	18649874
Peripheral blood	CD4⁺Foxp3⁺	Unchanged proportion	Impaired function	19036923
Peripheral blood	CD4⁺CD25^high^CD127^low/−^	Decreased proportion	—	21921095
Peripheral blood	CD4⁺CD25⁺Tim-3⁺Foxp3⁺	Decreased proportion	Impaired function	28478516
Synovial fluid	CD4⁺CD25^high^	Increased proportion	Normal function	12594850
Synovial fluid	CD4⁺CD25⁺	Increased proportion	Normal function, but impaired proliferative capacity	15807863
Peripheral blood and synovial fluid	CD4⁺CD25^high^	Decreased in peripheral blood, increased in synovial fluid	Normal function	15225369
Peripheral blood and synovial fluid	CD4⁺CD25⁺Foxp3⁺	Decreased in peripheral blood, increased in synovial fluid	—	18092263
Peripheral blood, synovial fluid, synovial membrane	CD4⁺CD25⁺/^high^CD127^low/−^	Higher proportion of Tregs in SF and SM compared to PB	SM Tregs: activated memory phenotype; PB Tregs: resting memory phenotype	24742142
Peripheral blood and synovial membrane	CD4⁺CD25^high^	Reduced in peripheral blood of active RA patients; higher in synovial fluid than PB in RA	Normal function	16571607

*PB* Peripheral blood, *SF* Synovial fluid, *SM* Synovial membrane

Changes in the inflammatory microenvironment, including hypoxia, excessive inflammation, and elevated ROS, likely contribute to Treg instability and impaired function in RA. For example, in the hypoxic synovial microenvironment of patients with RA, HIF-1α is activated in Tregs, and overexpressed HIF-1α binds to Foxp3, promoting its ubiquitination and degradation, thereby compromising Treg stability and suppressive function.^170,378^ Additionally, in collagen-induced arthritis mouse models, fibroblast-derived IL-6 induces the conversion of Tregs into Th17-like Tregs within the synovial membrane, which lose their immunosuppressive capacity and promote osteoclastogenesis.^379^ As mentioned previously, excessively high levels of ROS may impair Treg function. High levels of ROS at inflammatory sites in RA also oxidize PTEN, activating the downstream Akt/mTOR/STAT3 signaling pathway, upregulating SOCS3, and ultimately downregulating Foxp3 expression and destabilizing Tregs.^378,380^

In addition to exhibiting functional impairment and phenotypic instability, which hinder their ability to suppress aberrant inflammatory responses, Tregs contribute to RA progression via additional mechanisms. Under the influence of IL-1β, Tregs upregulate RANKL expression. Through RANKL–RANK signaling, Tregs promote the differentiation of osteoclasts.^381^ This process plays a critical pathogenic role in the destruction of bone associated with arthritis. Moreover, synovial Tregs express high levels of the tissue Treg marker ST2. These ST2⁺ Tregs compete for environmental IL-33, reducing eosinophil abundance and exacerbating disease severity.^382^

Several factors may also affect the abundance of synovial Tregs, further aggravating the imbalance between Tregs and proinflammatory cells. In patients with RA, Treg migratory capacity is impaired, possibly due to IL-6-mediated downregulation of G protein signaling modulator 2 (GPSM2) and decreased phosphorylation of vasodilator-stimulated phosphoprotein.^383,384^ Additionally, CD8⁺ T cells specific for self-antigen epitopes on apoptotic T cells can kill Tregs, disrupting the expansion of Tregs induced by anti-TNF therapy and contributing to resistance in a subset of patients.^385^

In summary, Tregs in RA may exhibit functional abnormalities and altered proportions, contributing to multiple pathogenic mechanisms of the disease (Fig. 6d). Future research should leverage emerging technologies to elucidate the molecular mechanisms underlying Treg dysregulation within the inflammatory microenvironment and explore strategies to restore Treg stability and immunosuppressive function, offering new therapeutic avenues for RA.

#### Tregs in T1D

T1D is the most prevalent autoimmune disease in humans and is caused by the immune system targeting insulin-producing β-cells, resulting in impaired insulin secretion and ineffective glucose utilization, ultimately leading to hyperglycemia.^386^ IPEX (immune dysregulation, polyendocrinopathy, enteropathy, X-linked syndrome) is a genetic disorder characterized by Treg dysfunction, and T1D is one of its core manifestations.^387^ Therefore, defects in Tregs may play a critical role in the pathogenesis of T1D. Studies in patients with T1D have also confirmed Treg dysfunction under disease conditions, characterized by a higher level of *Foxp3* TSDR methylation than in healthy controls and increased secretion of IFN-γ and IL-17, indicating an unstable Treg phenotype.^388–390^

The causes of Treg dysfunction in patients with T1D are partially understood and may be associated with mutations in genes related to Treg stability and function, such as *RARA*, *IL2RA*, *PTPN2*, *PTPN22* and *CTL4A-4*.^391–393^ In addition to genetic predisposition, recent studies have shed light on other potential mechanisms underlying Treg dysfunction in T1D. MicroRNAs (miRNAs) critically regulate immune homeostasis, and their dysregulation contributes to Treg dysfunction in T1D.^394^ For example, one study demonstrated that during the early stages of T1D in children, the increased strength of stimulation and costimulation signals mediated by miRNA-181a in T cells caused an increase in NFAT5 expression while inhibiting the induction of pTregs.^395^ Moreover, miR-142-3p was found to be abnormally upregulated in CD4⁺ T cells from both patients with T1D and NOD mice at disease onset. The increase in miR-142-3p suppresses Tet2 expression, disrupts the demethylated state of the Foxp3 locus, impairs Treg stability, and promotes autoimmune activation.^389^ HMGB1, passively released from damaged pancreatic β cells, is believed to play a key role in T1D pathogenesis by activating the immune system.^396^ In both NOD mice and patients with T1D, HMGB1 may impair Treg stability and suppressive function by activating the receptor for advanced glycation end products and TLR4, thereby promoting PI3K–Akt–mTOR signaling.^397^ Notably, HMGB1 blockade has been shown to prevent insulitis progression and reduce the incidence of diabetes.^397^ In addition, defective or dysregulated IL-2 and Nrp1 signaling,^398–400^ as well as excessive proinflammatory cytokines such as IL-6 and IFN-γ,^401^ can impair Treg differentiation and function, leading to an imbalance between Tregs and Teffs. Finally, hyperglycemia itself may further exacerbate Treg dysfunction (Fig. 6e).^402^

Currently, multiple clinical studies targeting Tregs have been initiated in T1D, including low-dose IL-2 administration and adoptive Treg transfer, both of which have shown some potential benefits.^403–406^ Future research should integrate genetic, epigenetic, metabolic, and inflammatory pathways to systematically uncover the key mechanisms underlying Treg dysfunction in T1D and develop more precise and effective therapeutic strategies.

### Tregs in nonautoimmune chronic inflammatory diseases

In response to harmful stimuli or environmental stress, tissues often undergo stress reactions and functional disturbances, leading to an intermediate state between tissue homeostasis and classical inflammation—referred to as para-inflammation. The primary role of para-inflammation is to promote adaptive adjustments and functional restoration of the tissue.^314^ However, if tissue dysfunction persists, para-inflammation may evolve into a chronic and pathological inflammatory state, which not only fails to restore homeostasis but may further exacerbate tissue damage and drive disease progression.^314,316^ This chronic low-grade inflammatory state is a feature of many diseases, including atherosclerosis, hypertension, obesity, type 2 diabetes, asthma, and neurodegenerative disorders. Tregs play a crucial role in these conditions, either by suppressing inflammation and promoting tissue repair to slow disease progression or, conversely, by contributing to disease development owing to functional impairment.

#### Tregs in atherosclerosis

Atherosclerosis is a chronic inflammatory disease triggered by hypercholesterolemia. It is characterized by the accumulation of oxidized low-density lipoprotein in the arterial wall and the formation of plaques enriched with immune cells.^407^ Recent clinical trials, such as CANTOS and COLCOT, have demonstrated that targeting inflammation can reduce cardiovascular events, underscoring the pivotal role of inflammation in atherogenesis.^408,409^ Both innate and adaptive immune responses, including those involving Tregs, are involved in the progression of atherosclerosis.

Accumulating evidence has shown that reduced Treg numbers are associated with exacerbated atherosclerosis. The depletion of Tregs through various approaches accelerates disease progression,^410,411^ whereas, adoptive transfer of Tregs into Apoe⁻/⁻ mice attenuates lesion development.^412^ In addition to suppressing inflammatory cells through classical immunosuppressive molecules such as IL-10, TGF-β, and CTLA-4, Tregs promote the enrichment of anti-inflammatory M2-like macrophages and enhance macrophage efferocytosis, thereby facilitating plaque regression and tissue repair.^413,414^ Clinically, a higher proportion and number of Tregs in atherosclerotic plaques and peripheral blood are positively correlated with plaque stability.^415^ These findings collectively suggest that functional Tregs confer atheroprotective effects.

However, Tregs within atherosclerotic plaques exhibit considerable heterogeneity and instability, with distinct phenotypes and functional profiles. For example, in advanced atherosclerotic mice, protective CXCR3⁺ Tregs are diminished and replaced by Th1-like Tregs expressing IFN-γ, T-bet, and CCR5. These Th1-like Tregs originate from functionally competent Tregs and exhibit impaired immunosuppressive function.^416,417^ A subset of Tregs may also transdifferentiate into Tfh-like cells that promote atherogenesis, characterized by the loss of Foxp3 expression and suppressive capacity.^418^ This phenotypic shift may be driven by increased cholesterol accumulation in Tregs due to a high-fat diet or oxLDL uptake, resulting in reduced IL-2 receptor expression and signaling, along with the upregulation of IL-6R and Bcl6.^418^ Th17-like Tregs have also been observed in mice with advanced atherosclerosis.^419^ Furthermore, in both patients and mouse models, ApoB-specific Tregs, which recognize atherosclerosis-associated antigens, gradually lose their Treg transcriptional identity and acquire proinflammatory features. These cells exhibit upregulated expression of transcription factors such as ROR-γt and T-bet, alongside increased inflammatory cytokine production, and display characteristics of effector and memory T cells.^420^ Notably, adoptive transfer of ApoB-specific Tregs into immunocompetent Apoe^⁻/⁻^ recipient mice failed to exert protective effects and instead aggravated atherosclerosis.^421,422^

The mechanisms underlying Treg instability and transdifferentiation into various subsets remain incompletely understood and may involve genetic predispositions, diet, TCR signaling, metabolic status, and the inflammatory microenvironment.^418,422–425^ Additionally, whether different Treg phenotypes are interrelated is still unclear. Future research should focus on strategies to stabilize Treg phenotypes in atherosclerosis, offering potential therapeutic and preventive avenues.

#### Tregs in hypertension

Hypertension is the leading risk factor for cardiovascular diseases, and the sustained activation of immune cells and chronic low-grade inflammation are closely associated with their development and progression.^426^ Among immune cells, CD4⁺ T cells—especially Th1, Th17, and Treg cells—play a central role. In patients and animal models of hypertension, an imbalance between proinflammatory Th1/Th17 cells and anti-inflammatory Tregs, along with increased levels of proinflammatory cytokines, has been observed.^426^ Multiple studies have demonstrated that adoptive transfer of Tregs or strategies to increase their abundance can effectively suppress blood pressure elevation and mitigate target organ damage in various animal models of hypertension.^427–429^ The Treg-derived anti-inflammatory cytokine IL-10 contributes to the preservation of cerebral blood flow responses and attenuates both systemic and cerebral inflammation in hypertension.^430^

The reduction in and dysfunction of Tregs in hypertension may be related to the actions of angiotensin II (Ang II). Ang II has been shown to upregulate C3aR and C5aR expression on Tregs, and through C3a/C3aR and C5a/C5aR signaling, it suppresses Foxp3 expression and impairs their immunosuppressive function.^431^ In addition, Ang II upregulates HDAC6 and induces the conversion of Tregs into proinflammatory Th1-like cells.^432^ Ang II also upregulates the expression of legumain (LGMN) in CD4⁺ T cells, which facilitates the degradation of TRAF6 and inhibits the NF-κB signaling pathway, thereby suppressing Treg differentiation while promoting Th1 polarization and enhancing IFN-γ production.^433^ Dietary factors such as high-salt and high-fat intake can also affect the differentiation balance of splenic Th17 cells and Tregs, influencing the development of hypertension.^324,325,434^ Although the precise roles and mechanisms underlying Treg dysregulation in hypertension remain to be fully elucidated, Tregs represent promising therapeutic targets for slowing hypertension progression.

#### Tregs in obesity

In the past few decades, the global prevalence of obesity has continued to rise, with some countries reporting that more than 50% of the adult population is affected.^435^ Adipose tissue is a dynamic organ distributed throughout the body, serving not only as an energy reserve but also as an endocrine organ that participates in whole-body glucose metabolism and responds dynamically to dietary changes and the body’s energy balance.^436,437^ The imbalance between caloric intake and energy expenditure in obese individuals leads to adipose tissue expansion and the onset of chronic low-grade inflammation. Notably, chronic low-grade inflammation and dysfunction of visceral adipose tissue (VAT) are key drivers of obesity-related metabolic abnormalities, such as insulin resistance and type 2 diabetes.^437,438^ The mechanisms by which obesity induces adipose tissue inflammation include increased ROS levels leading to oxidative stress,^439^ ER stress activating intracellular inflammatory pathways,^440^ free fatty acids activating inflammasomes and pattern recognition receptors,^438^ hypoxia and mechanical stress caused by excessive adipocyte expansion.^438,441^

VAT Tregs exhibit unique features in terms of transcriptomics, TCR repertoire, and especially high expression of the adipocyte transcription factor peroxisome proliferator-activated receptor gamma (PPARγ), which is a hallmark driver of their tissue-specific differentiation.^442^ Specific knockout of PPARγ in Tregs leads to a significant reduction in VAT Treg numbers and impaired insulin sensitivity.^442^ Additionally, the expression of ST2, the receptor for IL-33, is upregulated in VAT Tregs from both mice and humans.^443,444^ VAT Tregs are crucial for limiting VAT inflammation and maintaining metabolic health, whereas high-fat diets lead to a reduction in VAT Treg numbers, exacerbating VAT inflammation and insulin resistance.^445^ The mechanisms by which high-fat diets reduce VAT Tregs remain unclear but may involve pDC-mediated IFNα-dependent killing of VAT Tregs, GATA3 downregulation that impairs ST2⁺ Treg differentiation, and disrupted cholesterol homeostasis that limits ST2⁺ Treg clonal expansion.^49,446,447^ IL-33 can induce VAT-Treg proliferation, and in obese mice or high-fat diet models, IL-33 can restore VAT-Treg numbers and improve metabolism.^443,444,448^

Further research has revealed notable heterogeneity and sex differences in VAT Tregs.^48,49^ Two major types of mature Tregs reside in mouse VAT: IL-33-dependent PPARγ^+^ST2^+^ VAT Tregs and T-bet^+^CXCR3^+^ VAT Tregs. The former predominates in males, whereas the latter is more abundant in females. These two Treg subsets play distinct roles in suppressing VAT inflammation and maintaining glucose homeostasis. In both male and female mice, the absence of CXCR3^+^ Tregs leads to exacerbated VAT inflammation without affecting glucose tolerance, whereas the depletion of ST2^+^ Tregs impairs glucose tolerance in male mice but has no significant effect on VAT inflammation.^49^ Inflammation itself can influence VAT Tregs, as inflammatory mediators such as IL-6, TNF, and CCL2 can promote the recruitment and transformation of ST2^+^ VAT Tregs. Estrogen, which has anti-inflammatory effects, may explain why ST2^+^ VAT Tregs are more enriched in male mice.^48^

However, VAT Tregs do not always play a protective role. For example, in Treg-specific PPARγ knockout mice, depletion of VAT Tregs improved glucose tolerance and insulin sensitivity in aged mice.^449^ Additionally, knockout of the transcription factor Blimp-1 in Tregs leads to a reduction in the number of ST2^+^KLRG1^+^ Tregs that secrete IL-10 in adipose tissue but unexpectedly enhances insulin sensitivity and reduces obesity development.^450^ Thus, while the role of VAT Tregs has been partially elucidated, we have yet to fully elucidate how VAT Tregs maintain adipose tissue homeostasis and normal function. Thus, further exploration of their specific mechanisms is warranted.

#### Tregs in nonalcoholic fatty liver disease (NAFLD) and nonalcoholic steatohepatitis (NASH)

NAFLD and NASH are among the most common causes of chronic liver disease and are closely associated with metabolic dysfunctions such as obesity. NAFLD is characterized primarily by hepatic steatosis, whereas NASH is its progressive form, involving inflammation and fibrosis.^451^ In the livers of patients with NASH, Treg numbers increase with increasing Areg and αvβ8 expression, which exacerbates liver fibrosis via the EGFR signaling pathway and promotes TGF-β activation, respectively.^452,453^ Treg-derived Areg also enhances hepatic gluconeogenesis, contributing to glucose metabolism disorders.^452^ Moreover, neutrophils are markedly increased in NASH livers, where they promote mitochondrial respiration via NETs to drive the differentiation of naïve CD4⁺ T cells into Tregs. These Tregs accumulate during the progression from NASH to HCC, ultimately contributing to tumor development by suppressing cancer immunosurveillance.^454^ In addition, IL-33 derived from aged hepatic stellate cells activates ST2⁺ Tregs, driving CD8⁺ T-cell exhaustion and promoting the progression of obesity-associated hepatocellular carcinoma.^455^ However, Tregs may also play a protective role, and adoptive transfer of Tregs has been shown to alleviate liver inflammation in experimental models.^456,457^ These conflicting findings reflect the dual roles of Tregs in NASH—both in promoting fibrosis and inhibiting inflammation.

#### Tregs in neurodegenerative diseases

Neurodegenerative diseases, such as Alzheimer’s disease (AD), Parkinson’s disease (PD), and amyotrophic lateral sclerosis (ALS), are characterized by the progressive loss of neurons and neurological function. Inflammation in the central nervous system, driven by microglia and astrocytes, may play a critical role in the pathological progression of these diseases.^458^ In addition to innate immune cells, adaptive immune cells, particularly Tregs, have been increasingly recognized as important players in neurodegenerative diseases.^459^ In addition to expressing classical immunosuppressive molecules such as IL-10 and TGF-β, Tregs can induce the transition of microglia from a proinflammatory M1 state to an anti-inflammatory M2 state and promote the shift of astrocytes from a neurotoxic A1 subtype to a neuroprotective A2 subtype, thereby suppressing inflammation.^459–461^ A growing body of evidence suggests that in addition to their immunomodulatory functions, Tregs possess reparative capabilities, including direct neuroprotection, promotion of remyelination and neural regeneration, and vascular repair.^52,459^

However, transient depletion of peripheral Tregs in the middle stage of AD can alleviate disease symptoms. This is likely due to Treg-mediated immunosuppression impairing the “gateway” function of the choroid plexus. Treg depletion restores this gateway, facilitating the recruitment of various immunomodulatory cells, including Tregs themselves, into the central nervous system.^462^ Furthermore, Treg dysfunction has been observed in certain neurodegenerative diseases. For example, patients with rapidly progressing ALS exhibit reduced Treg numbers and diminished Foxp3 expression, resulting in impaired immunosuppressive function.^463^ In aging individuals, Tregs show a decreased ability to promote oligodendrocyte differentiation and remyelination, which limits their therapeutic potential for remyelination in older patients.^464^ Therefore, Tregs may play distinct roles in different stages of neurodegenerative diseases. It is necessary to further elucidate their dynamic changes and functional characteristics during disease initiation and progression to provide a theoretical basis for the development of precise immunotherapeutic strategies.

#### Tregs in asthma

Asthma is a common chronic inflammatory airway disease characterized by an abnormal type 2 immune response mediated by Th2 in response to commonly inhaled aeroallergens such as house dust mites, pollen, and animal dander. Typical features of asthma include airway narrowing and eosinophilia in sputum.^465^ Tregs play a key role in suppressing airway inflammation.^466,467^ Imbalances between Th cells and Tregs are closely associated with asthma development, and adoptive transfer of Tregs or pharmacological expansion of Treg populations has been shown to alleviate asthma symptoms.^466–469^

Tregs in patients with asthma typically exhibit functional impairments. Notably, allergens and IL-6 can induce the expression of Notch4 in Tregs, which disrupts their immunosuppressive function via activation of the Wnt and Hippo signaling pathways. Moreover, these Notch4⁺ Tregs produce GDF15, which activates ILC2s and thereby exacerbates inflammation.^470^ The stability and function of Tregs are also regulated by IL-33/ST2 signaling.^471,472^ IL-33 exposure prompts pulmonary Tregs to increase GATA3 and ST2 expression, produce type 2 cytokines, and lose their ability to suppress Teffs, thereby promoting type 2 airway inflammation.^472^ Notably, after puberty, the prevalence of asthma is lower in males than in females, possibly due to androgen signaling, which suppresses IL-33 secretion and ST2 expression on Tregs, increasing their suppressive capacity and phenotypic stability and thereby attenuating allergic airway inflammation.^473^ In addition, elevated levels of lyso-phosphatidylglycerol 18:0 (LPG 18:0) observed in patients with asthma have been shown to reduce Foxp3 protein levels via SIRT1-mediated deacetylation, impairing the differentiation of naïve CD4⁺ T cells into Tregs and weakening their immunosuppressive activity.^474^ Several environmental factors linked to asthma pathogenesis also influence Treg differentiation and function. Cigarette smoke exposure increases neutrophil counts and the formation of NETs, which enhances DC antigen presentation, promotes Th17 differentiation, and suppresses Treg generation.^475^ Ambient air pollution exposure increases methylation of the *Foxp3* gene, thereby impairing Treg function and worsening asthma symptoms.^476^ PM2.5 induces HIF-1α and glutamic-oxaloacetic transaminase 1 (GOT1) expression via activation of AHR, further promoting Th17 differentiation and inhibiting Treg development.^477^ These findings underscore the critical role of Treg deficiency and dysfunction in asthma pathogenesis and provide new therapeutic targets.

### Treg-targeted therapies for inflammatory diseases

**Fig. 7:**
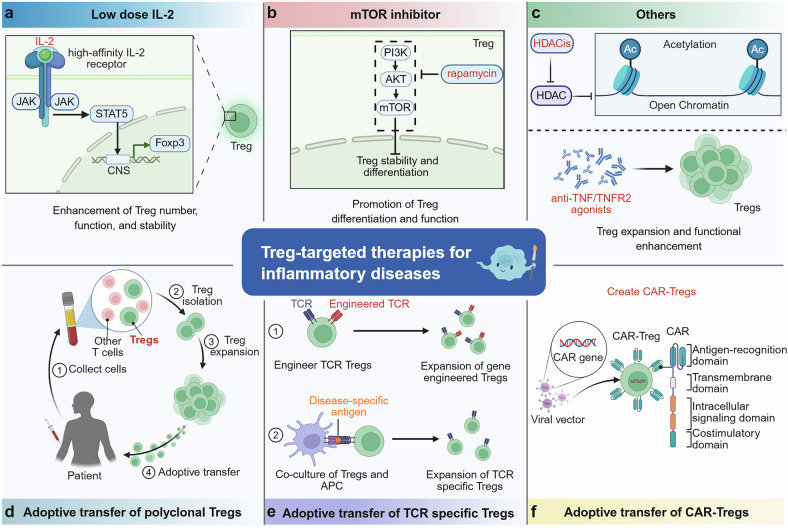
Targeting Tregs for the treatment of inflammatory diseases. Current therapeutic strategies targeting Tregs in inflammatory diseases focus primarily on either enhancing endogenous Tregs or adoptively transferring exogenous Tregs. **a** Low-dose IL-2 promotes the expression and stability of Foxp3 by activating the JAK–STAT5 signaling pathway. **b** mTOR inhibitors facilitate Treg differentiation and help maintain Treg functional stability by suppressing the mTOR signaling pathway. **c** HDACis and anti-TNF/TNFR2 agonists can also promote the expansion and functional enhancement of endogenous Tregs. **d** In polyclonal Treg adoptive transfer therapy, Tregs are isolated from patients, expanded ex vivo, and reinfused into patients to increase the number of Tregs in vivo. **e** TCR-specific Tregs are generated by introducing disease-specific TCRs or coculturing with APCs loaded with disease-relevant antigens, yielding antigen-specific Tregs with enhanced targeting precision. **f** CAR-Tregs express CARs that recognize disease-associated surface antigens, enabling MHC-independent targeting and potentially broader clinical applicability. This figure was created with Biorender.com

Functionally competent Tregs play pivotal roles in suppressing aberrant inflammatory responses and mitigating the progression of inflammatory diseases. However, in many chronic inflammatory conditions, Tregs often exhibit functional impairment and an imbalanced ratio relative to proinflammatory T cells, contributing to disease onset and progression.^8,9^ Consequently, therapeutic strategies aimed at expanding or enhancing Tregs have garnered increasing attention for their potential to suppress inflammation and ameliorate disease severity.^10,478–480^ These approaches include increasing the number of endogenous Tregs and the ACT of exogenous Tregs (Fig. 7).

#### Expansion and enhancement of endogenous Tregs

##### Low-dose IL-2 therapy

Low-dose IL-2 therapy represents the most promising strategy for expanding endogenous Tregs (Fig. 7a). IL-2, a T-cell growth factor, functions by binding to IL-2R, which is composed of three subunits: IL-2Rα, IL-2Rβ, and IL-2Rγ. The high-affinity IL-2 receptor, formed by all three subunits, is constitutively expressed on Tregs, rendering them highly sensitive to IL-2 and enabling them to efficiently outcompete other cells for the limited amount of IL-2 available in vivo.^481^ The IL-2–STAT5 signaling axis is essential for Treg differentiation, survival, and functional competence.^22,59^ IL-2 activates JAK1 and JAK3, inducing the phosphorylation and activation of STAT5, which then binds to the promoter and CNS of the *Foxp3* gene, facilitating stable Foxp3 expression.^25,59,481^ In the absence of IL-2, Tregs fail to survive in peripheral lymphoid organs.^400^ Preclinical studies have demonstrated that exogenous IL-2 administration yields favorable outcomes in chronic inflammatory disease models.^481^ Several clinical trials are currently underway to assess the safety and efficacy of IL-2 in treating inflammatory diseases such as T1D,^403,404^ SLE,^482^ and MS,^483^ and have shown encouraging preliminary results. However, notably, IL-2 can also promote the expansion and activation of proinflammatory T-cell subsets, including effector and memory T cells.^481^ Therefore, low-dose IL-2 therapy is designed to preferentially expand Tregs while minimizing undesired effects on other immune cells.^481^ In addition, researchers have developed more Treg-selective immunotherapeutic strategies to minimize side effects, such as IL-2 muteins and PEGylated IL-2.^484,485^

##### mTOR inhibitor therapy

mTOR inhibitors represent another promising approach to expand and enhance Tregs (Fig. 7b). The mTOR signaling pathway, comprising the mTORC1 and mTORC2 complexes, plays a multifaceted role in immune cells by regulating cellular metabolism, proliferation, differentiation, and effector functions.^486^ The role of mTOR signaling in Tregs is complex and somewhat paradoxical. Appropriate levels of mTOR activity are required for Tregs to exert their immunosuppressive functions, whereas dysregulated mTOR signaling can compromise Treg stability.^70,72^ Notably, mTOR signaling negatively regulates the in vitro induction of iTregs.^487^ Therefore, mTOR inhibition in the context of inflammatory diseases may contribute to disease control, at least in part, by increasing the Treg frequency and function. For example, in patients with RA, combined treatment with low-dose rapamycin and conventional immunosuppressants increases the proportion of peripheral Tregs and reduces disease activity compared with immunosuppressants alone, without significant adverse effects.^488^ Similarly, a clinical trial of low-dose rapamycin in patients with ALS revealed a trend toward increased Treg numbers in the treatment group compared with the placebo group.^489^ Moreover, mTOR inhibition has been employed during in vitro expansion of polyclonal or engineered Tregs to stabilize their phenotype and maintain their suppressive potential.^490^

##### Other strategies to enhance Tregs

In addition to low-dose IL-2 and mTOR inhibitors, several other pharmacological strategies have been explored to expand or enhance endogenous Tregs for the treatment of inflammatory diseases (Fig. 7c). Proper histone acetylation is essential for the expression of Foxp3 in Tregs.^60^ Accordingly, histone deacetylase inhibitors (HDACis) can upregulate Foxp3 expression and increase Treg numbers and functionality and have shown therapeutic benefits in various inflammatory conditions, including RA,^491^ IBD,^340^ and asthma.^492^ Moreover, TNF receptor 2 (TNFR2) expression is critical for Treg function and stability. TNFR2-deficient Tregs display hypermethylation of the *Foxp3* TSDR region, leading to impaired stability.^493^ Therapeutic strategies targeting this pathway, including anti-TNF agents and TNFR2 agonists, have been shown to increase Treg frequency and suppressive function, thereby alleviating symptoms in inflammatory disease.^494–496^

#### Adoptive transfer of exogenous Tregs

ACT therapy using exogenous Tregs has shown promising outcomes in the treatment of various inflammatory diseases. This approach includes the transfer of in vitro-expanded polyclonal Tregs, TCR-specific Tregs, and chimeric antigen receptor (CAR)-engineered Tregs.^10,478,479^

##### Adoptive transfer of polyclonal Tregs

In preclinical models of chronic inflammatory diseases, adoptive transfer of exogenous Tregs has been shown to reduce proinflammatory immune cell infiltration and ameliorate disease severity. On the basis of these findings, multiple clinical trials are currently underway to assess the safety and efficacy of polyclonal Treg-based ACT in inflammatory diseases such as T1D,^403,405,406^ IBD,^497^ and ALS.^498^ For example, in one study, autologous Tregs were isolated from patients with T1D, expanded ex vivo, and reinfused, demonstrating favorable safety and tolerability profiles, with some patients exhibiting delayed disease progression.^405^ Ensuring the purity, functionality, and phenotypic stability of Tregs during isolation and subsequent expansion is critical for successful Treg-based ACT.^10,478^ Notably, a clinical trial of adoptive Treg transfer in T1D revealed that lower fold expansion of Tregs was associated with better C-peptide preservation, suggesting that excessive in vitro expansion may not necessarily improve therapeutic efficacy.^406^ A key challenge following ACT is ensuring the long-term persistence of Tregs in vivo. In treated patients with T1D, although the transferred Tregs retained a stable phenotype, their frequency in the peripheral blood declined rapidly, with more than 75% disappearing from the circulation within 90 days.^405^ To address this, some researchers have proposed the coadministration of low-dose IL-2 to increase the survival and expansion of transferred Tregs. However, this approach may also promote the proliferation of other immune cell subsets, including CTLs, which could compromise therapeutic outcomes.^403^ Subsequent studies could optimize therapeutic strategies by using more Treg-specific IL-2–based agents (Fig. 7d).

##### Adoptive transfer of TCR-specific Tregs

Compared with polyclonal Tregs, antigen-specific Tregs can more efficiently home to target tissues and exert therapeutic effects with a smaller number of cells.^478^ However, the frequency of naturally occurring antigen-specific Tregs in peripheral blood is extremely low, posing significant challenges to their direct isolation and subsequent expansion to therapeutic doses.^10^ TCR-specific Tregs are typically generated through two main approaches: one involves boosting recipient Tregs with donor-derived APCs, and the other employs genetic engineering to introduce recombinant TCRs into Tregs, thereby producing TCR-engineered, antigen-specific Tregs (Fig. 7e).^10^ Preclinical studies have demonstrated the efficacy of TCR-specific Tregs in various disease models. For example, amyloid β–specific human Tregs have been used to treat AD;^499^ engineered Tregs targeting islet antigens have been explored for T1D;^500^ myelin oligodendrocyte glycoprotein (MOG)-specific Tregs have shown efficacy in EAE;^501^ and type II collagen-specific Tregs have been tested in RA models.^502^ Furthermore, upon TCR activation, Tregs upregulate the expression of cytosolic and membrane-associated reductases. Leveraging this feature, researchers have developed redox-sensitive IL-2 nanoparticles that release IL-2 in response to the reductive environment triggered by specific TCR activation, thereby enhancing the precision and efficacy of TCR-specific Treg-based therapies.^503^ Despite these advances, limited knowledge of Treg-specific TCR repertoires and their cognate peptide antigens remains a major bottleneck in the development of TCR-specific Tregs. Emerging technologies such as single-cell TCR sequencing hold promise for identifying novel Treg-specific TCRs and broadening the application of this targeted immunotherapy approach.^504^

##### Adoptive transfer of CAR-Tregs

CAR-Tregs represent another form of engineered regulatory T cells. CARs are synthetic receptors that typically consist of an antigen-recognition domain, a transmembrane domain, an intracellular signaling domain that mimics TCR activation, and costimulatory domains such as CD28 or 4-1BB (Fig. 7f).^505^ Unlike TCRs, which are MHC restricted and recognize peptide antigens presented on MHC molecules, CARs recognize antigens in an MHC-independent manner.^10,478^ This allows broader patient applicability. However, CAR activation typically requires increased antigen density and is limited to recognizing extracellular antigens.^478^ Various CAR-engineered Treg constructs have been developed for the treatment of chronic inflammatory diseases. For example, Foxp3-expressing CAR-Tregs specific for CD19 (Foxp3-CD19 CAR-Tregs) have been shown to suppress B-cell proliferation, function, and autoantibody production in SLE.^506^ Insulin B chain 10–23 peptide–specific CAR-Tregs (InsB-g7 CAR-Tregs) have been used in models of T1D.^507^ IL-23 receptor-targeted CAR-Tregs (IL-23R CAR-Tregs) and flagellin-targeted CAR-Tregs (FliC CAR-Tregs) have demonstrated efficacy in colitis models.^508,509^ CAR-Tregs targeting myelin oligodendrocyte glycoprotein (CARαMOG-Tregs) have been applied in EAE.^790^ Carcinoembryonic antigen (CEA)-specific CAR-Tregs have been evaluated for asthma treatment, given the high expression of CEA on lung epithelial cells.^510^

Despite promising results, several challenges remain for CAR-Treg–based therapies in inflammatory diseases. First, most CAR Treg studies have employed second-generation CAR constructs. To fully harness the therapeutic potential of CAR-Tregs, continually refining CAR design, including the identification of optimal target antigens and the optimization of various structural domains, is essential.^511^ Second, as with all adoptive Treg therapies, ensuring the in vivo stability and persistence of CAR-Tregs is crucial for achieving long-term efficacy. Tonic signaling—constitutive CAR signaling in the presence or absence of ligand engagement—particularly associated with 4-1BB costimulatory domains—has been shown to impair CAR-Treg stability and function. Transient inhibition of the mTOR pathway can rescue 4-1BB CAR-Tregs from tonic signal-induced dysfunction.^512^ Finally, whether CAR-Tregs may cause cytokine release syndrome, as observed in CAR-T-cell therapies for cancer, remains a critical safety concern that warrants further investigation.^513^

## Conclusions and perspectives

As critical regulators of the immune system, Tregs play a key role in maintaining immune homeostasis and are involved in the development and progression of various diseases. Tregs exhibit a “double-edged sword” nature: within the TME, they often inhibit antitumor immunity and promote immune evasion, thereby facilitating tumor progression; however, they are also essential for suppressing excessive immune responses and preventing autoimmunity. Therefore, the role of Tregs is highly context dependent, and their functional complexity and dynamism make them pivotal nodes in immune regulation.

Within the TME, Tregs are markedly expanded and infiltrated, exhibiting potent immunosuppressive capabilities. They suppress effector T-cell activity, interfere with antigen presentation by APCs, and modulate components such as M2-like TAMs and CAFs,^34,123,126,128^ thus shaping an immunosuppressive milieu conducive to tumor growth. Tregs can also directly promote tumor metastasis.^121,122^ TI-Tregs show strong environmental adaptability, maintaining their stability under complex stimuli. This environmental adaptability is a key basis for their role in promoting tumor progression and mediating resistance to antitumor therapies. Combining Treg-targeting strategies with existing therapies holds promise for enhancing therapeutic efficacy and overcoming immunosuppressive barriers. Future studies should focus on elucidating the regulatory mechanisms governing Treg accumulation and function in tumors and developing intervention strategies with improved specificity and safety for clinical translation.

Conversely, in autoimmune diseases (e.g., RA, SLE, T1D, and IBD) and chronic inflammatory conditions (e.g., atherosclerosis, obesity, and asthma), Tregs exert protective effects by dampening hyperactive immune responses, promoting inflammation resolution, and facilitating tissue repair.^9^ A reduction in Treg number or proportions, as well as phenotypic instability and functional impairment, is often closely associated with disease onset and exacerbation. Thus, strategies aimed at increasing Treg numbers or enhancing their suppressive function represent promising therapeutic approaches for various autoimmune and inflammatory diseases.

Currently, multiple clinical studies are evaluating therapeutic strategies targeting Tregs, such as anti-CD25-induced Treg depletion and anti-CCR4 and CCR8 antibodies to inhibit Treg recruitment for cancer treatment;^7^ low-dose IL-2 therapy and adoptive Treg transfer therapy are being explored for the treatment of autoimmune diseases.^478,481^ Although these strategies remain exploratory and require further optimization in terms of safety and specificity, preliminary results suggest that disease microenvironment-tailored modulation of Tregs holds great therapeutic potential.

In recent years, accumulating evidence has revealed that Tregs are not static but rather exhibit high plasticity and functional instability.^13^ This instability poses both challenges and opportunities. In cancer, selectively inducing Treg instability or fragility may help alleviate immunosuppression and enhance antitumor immune responses. In inflamed tissues, stabilizing the identity and function of Tregs may restore or enhance their immunoregulatory capacity, thereby improving disease outcomes. Therefore, elucidating the mechanisms that govern Treg stability or destabilization in specific disease microenvironments and exploring precise modulation strategies have become key frontiers in immunotherapy research. The ultimate goal is to provide safer and more precise therapeutic strategies without impacting peripheral immune tolerance.

The rapid advancement of cutting-edge technologies provides strong support for achieving this goal. High-throughput techniques such as single-cell RNA sequencing, cytometry by time-of-flight, ATAC-seq, and spatial transcriptomics and proteomics allow researchers to analyze Treg heterogeneity and dynamic changes across tissues and disease contexts at unprecedented resolution. These tools reveal new Treg subsets, regulatory networks, and functional trajectories. Moreover, gene-editing technologies such as CRISPR/Cas9 enable precise functional validation of key targets both in vitro and in vivo. Furthermore, the development of nanoparticles and tissue-specific antibodies offers the potential for precise modulation of Tregs at the subpopulation or tissue level.

Future research should focus on systematically deciphering the dynamic regulatory mechanisms of Tregs in diverse disease settings, specifically in the following areas:

(1) Integrating multiomics data (e.g., single-cell transcriptomics, epigenomics, metabolomics, and spatial omics) with functional assays to construct comprehensive regulatory networks governing Treg development, homeostasis, and plasticity and to identify key regulators and signaling pathways;

(2) Elucidating the interplay between metabolic states, epigenetic modifications, and cytokine signaling in driving Treg fate decisions and functional heterogeneity under pathological conditions;

(3) Developing targeted delivery systems tailored to the lesion microenvironment to achieve tissue- or context-specific Treg modulation while avoiding off-target effects on peripheral Tregs;

(4) Prospective clinical studies should be conducted to evaluate the long-term efficacy and safety of Treg-targeted therapies across different disease types, ensuring improved therapeutic outcomes without inducing immune-related adverse effects or disrupting the systemic immune balance.

In conclusion, Tregs are indispensable immunoregulatory players across a wide spectrum of diseases. By broadening our understanding of their biological mechanisms and leveraging precise modulation technologies, we may achieve transformative breakthroughs in both cancer immunotherapy and the treatment of autoimmune and inflammatory disorders.
